# Prevalence of Social Media Addiction and Associations With Usage Patterns, Burnout, and Health Conditions Among Medical Trainees in China: Cross-Sectional Study

**DOI:** 10.2196/75675

**Published:** 2026-05-04

**Authors:** Zexu Guan, Ni Tang, Guoshuai Luo, Xiao Zhang

**Affiliations:** 1 School of International Politics and Communication, Beijing Language and Culture University, Beijing, China; 2 Peking University Sixth Hospital, Huayuanbei Road 51, Haidian District, Beijing, 100191, China, 86 01082805307; 3 Peking University Institute of Mental Health, Beijing, China; 4 NHC Key Laboratory of Mental Health, Peking University, Beijing, China; 5 National Clinical Research Center for Mental Disorders, Peking University Sixth Hospital, Beijing, China; 6 Tianjin Anding Hospital, Tianjin, China

**Keywords:** social media addiction, SMA, Chinese medical trainees, occupational burnout, social media usage time, U-shaped relationship

## Abstract

**Background:**

Medical residency is a demanding training stage characterized by high levels of stress and burnout. As digital natives, current medical trainees (ie, residents) are frequent users of social media; however, little is known about how their personal (nonprofessional) use relates to burnout and social media addiction (SMA).

**Objective:**

This study aims to characterize the prevalence of SMA among Chinese medical trainees and explore its complex relationships with social media use patterns, occupational burnout, and related risk and protective factors.

**Methods:**

A nationwide cross-sectional survey was deployed through Wenjuanxing and disseminated via WeChat between August 29 and September 10, 2024. Data included demographics, physical and psychiatric health history, work variables (eg, training year and night shifts), personality traits, and social media use. SMA was assessed using the Bergen Social Media Addiction Scale. Logistic regression was performed to identify predictors of addiction, and mediation and moderation analyses were conducted to clarify the role of occupational burnout.

**Results:**

Of 3621 medical trainees, 211 (5.8%) met the criteria for SMA (Bergen Social Media Addiction Scale ≥24, indicating addiction). Second-year medical trainees reported the highest addiction prevalence (92/1159, 7.9%). Logistic regression analysis revealed that higher burnout (odds ratio [OR] 1.41, 95% CI 1.23-1.62; *P*<.001), longer daily use (OR 1.39, 95% CI 1.23-1.56; *P*<.001), physical health problems (OR 1.56, 95% CI 1.13-2.16; *P*=.006), and psychiatric history (OR 2.00, 95% CI 1.41-2.84; *P*<.001) significantly increased the odds of addiction, whereas conscientiousness was protective (OR 0.92, 95% CI 0.86-0.99; *P*=.02). Social media use showed significant U-shaped associations with burnout, physical health problems, psychiatric history, personality characteristics, and mental health outcomes. For example, medical trainees using social media 1 hour or less (104/404, 25.7% with psychiatric history) and more than 4 hours daily (97/419, 23.2% with psychiatric history) both had higher risk profiles than moderate users. Mediation analysis showed that occupational burnout explained 28.1% of the effect of psychiatric history and 29.6% of the effect of physical health problems on addiction risk.

**Conclusions:**

This large-scale survey provides the first systematic characterization of SMA among Chinese medical trainees and elucidates its associated risks and protective factors. Burnout consistently emerged as a key and pervasive predictor of SMA, functioning both as an independent risk factor and as a mediator amplifying the impact of health-related vulnerabilities. Moreover, the findings highlight that both minimal and excessive daily social media use may signal distinct behavioral manifestations of distress, potentially reflecting different clinical phenotypes: digital disengagement under acute stress versus compulsive engagement driven by chronic burnout. Notably, while mental health symptoms exhibited U-shaped associations with usage, SMA risk increased progressively with daily duration. These results underscore the need for interventions that extend beyond simply monitoring usage duration, emphasizing strategies to reduce burnout and enhance the overall well-being of medical trainees.

## Introduction

Social media has become deeply embedded in the everyday lives of young adults and is increasingly intertwined with medical education, communication, and professional identity formation [1]. A recent systematic review of social media in undergraduate medical education showed that these platforms are now widely used by medical learners for communication, knowledge exchange, and informal learning, while also raising concerns regarding professionalism and potential adverse effects on mental and physical well-being [2]. However, growing evidence suggests that problematic or addictive patterns of social media use are associated with depression, anxiety, sleep disturbances, and poorer mental well-being, as highlighted in recent reviews and large-scale studies [3].

Social media addiction (SMA) is characterized not only by excessive time spent online but also by withdrawal symptoms (eg, irritability when access is restricted) and functional impairment in daily or occupational domains [4,5]. Despite growing evidence linking SMA to adverse mental health outcomes in medical learner populations, there remains limited comprehensive evidence on its prevalence, determinants, and potential mechanisms among medical trainees in China, a population uniquely vulnerable to chronic stress [6]. Given the rapid digitalization and widespread integration of social media into daily life in China [7], understanding SMA in this context is particularly important.

Medical trainees face a significant transition from an educational environment to clinical scenarios, including long working hours, frequent night shifts, high patient loads, and continuous exposure to tasks that demand substantial physical and emotional effort, as well as constant engagement with complex clinical situations [8]. Under these pressures, medical trainees may be particularly vulnerable to problematic social media use as a maladaptive coping strategy, which has been linked—along with poor work-life balance—to increased stress, depression, and burnout [9]. In parallel, prolonged work-related stress is a well-established driver of occupational burnout among medical trainees [10-14], characterized by a triad of symptoms: emotional exhaustion, depersonalization (cynicism), and a reduced sense of personal accomplishment [15]. However, the role of burnout in the development of SMA remains unclear, particularly whether it functions as an independent predictor or as a mediator linking vulnerability factors to addictive social media use.

To address these gaps, we conducted a data-driven, nationwide cross-sectional survey of Chinese medical trainees. This study aimed to (1) estimate the prevalence of SMA and identify associated risk and protective factors, including occupational burnout, health conditions (both physical and mental), and usage patterns; (2) characterize patterns of social media use (eg, daily usage time and number of platforms); and (3) examine the direct and indirect roles of burnout in the development of SMA.

## Methods

### Study Design and Participants

This study used a cross-sectional survey design to explore the factors contributing to SMA among medical trainees in China. Data were collected using an anonymous online questionnaire. A completed CHERRIES (Checklist for Reporting Results of Internet E-Surveys) checklist is provided in Checklist 1 to ensure the transparency and reproducibility of the e-survey results.

### Medical Training Pathway in China

In this study, the medical trainees were residents enrolled in China’s standardized residency training (SRT) program. They had completed a 5-year bachelor’s degree in medicine. Participants represented three common SRT pathways: (1) social track (independent medical students entering residency directly after undergraduate training), (2) integrated master’s track (students completing a coordinated master’s degree and residency program), and (3) other track (individuals with atypical educational routes, eg, PhD holders who subsequently entered residency).

### Survey Development and Pilot Testing

The questionnaire was developed by experienced psychologists and psychiatrists, drawing on validated instruments commonly used in mental health and behavioral research. The final questionnaire consisted of 36 major questions and 84 items, including matrix subitems. It had four sections: (1) basic information (15 major questions; eg, demographics and physical and psychiatric health conditions), (2) workload and lifestyle factors (9 major questions; eg, number of night shifts and daily social media use), (3) mental health status (3 major questionnaires), and (4) psychological characteristics (9 major questions; eg, personality traits and occupational burnout). The draft survey was pilot-tested with 20 graduate students to ensure clarity, usability, and technical functionality. Minor revisions were made based on the feedback.

### Survey Content and Completion

Respondents were required to complete all 80 core items before submission to ensure data completeness. The 4 noncore items were optional and included preferred social media influencers, positive experiences during residency training, negative experiences during residency training, and suggestions for educational or health authorities. The average completion time was 495 (SD 966) seconds (approximately 8 min).

### Survey Dissemination

The online survey was hosted by Wenjuanxing, the largest online survey platform in China. Recruitment was conducted via WeChat, the most widely used social media platform in China. Medical trainees could directly access and complete the survey by clicking the Wenjuanxing link on WeChat. The survey link was disseminated through professional groups and residency trainee networks. Data were collected for 2 weeks (from August 29, 2024, to September 10, 2024).

### Eligibility and Data Quality Assurance

Medical trainees enrolled in SRT programs were eligible. One mandatory item required participants to report their specialty, and responses outside the national residency catalog were excluded during data cleaning. To ensure authenticity, cookies were used to prevent duplicate entries, and questionnaires with implausibly short completion times (<2 min) were excluded. No statistical weighting or propensity score adjustments were applied because the sample size was sufficiently large and the sex distribution was balanced. Furthermore, as all mandatory items were required for submission, the final dataset contained no missing values.

### Ethical Considerations

This study was conducted in accordance with the Declaration of Helsinki and was approved by the ethics committee of Tianjin Anding Hospital (2023‐27). The informed consent form was presented on the first page of the online questionnaire, and only participants who checked the consent box were instructed to complete the remainder of the questionnaire. All mandatory questions were nonidentifiable and contained no items that could be used to trace participants. Participants who completed the survey received monetary compensation of 5 RMB (<US $1), which was considered unlikely to introduce response bias.

### Data Collection and Instruments

Social media use was assessed based on the type of platform used and the average daily duration of use. SMA was evaluated using the Bergen Social Media Addiction Scale (BSMAS), a brief self-report instrument derived from the component model of behavioral addiction. The BSMAS consists of 6 items assessing core addiction components (salience, mood modification, tolerance, withdrawal, conflict, and relapse), each rated on a 5-point Likert scale (1=“very rarely” to 5=“very often”), yielding a total score of 6 to 30 points. The scale has demonstrated good reliability and validity in Chinese populations, and a cutoff score of 24 or more was used to indicate probable SMA [16,17]. Importantly, a large meta-analysis of the prevalence of SMA across 32 countries reported that all included epidemiological studies used the BSMAS framework, including the original and translated versions, supporting its widespread adoption and cross-study comparability [18].

In addition to social media–related information, the questionnaire covered basic information such as age, sex, financial burden, physical health problems, psychiatric history, and prior psychological treatment. Physical health problems, psychiatric history, prior psychological treatment, and financial burden were assessed using binary (yes or no) self-report items. Participants were asked whether they had any chronic physical health conditions requiring long-term attention, had ever been diagnosed with mental health conditions such as anxiety or depression, had received psychological counseling or psychotherapy, and had family-related financial responsibilities (ie, providing financial support to parents or other family members or supporting children). For all items, a “yes” response indicated the presence of the corresponding condition or experience. The exact wording of all corresponding questionnaire items is provided in Multimedia Appendix 1.

Work-related variables included training year, average monthly night shifts, and occupational burnout, with burnout assessed on a 5-point Likert scale (1=“no burnout” to 5=“extremely high burnout”). Personality traits were measured using the Chinese version of the 10-Item Personality Inventory, which has demonstrated good reliability in assessing the Big Five personality dimensions in Chinese populations [19]. Mental health outcomes included depressive symptoms, anxiety, and sleep problems, which were assessed using the Patient Health Questionnaire-9, the Generalized Anxiety Disorder-7 scale, and the Insomnia Severity Index, respectively.

### Statistical Analysis

#### Descriptive Statistics

Descriptive statistics were used to summarize participants’ basic characteristics, work variables, personality traits, and social media usage. This step provided a clear picture of the key attributes of the sample and a foundation for further analysis. As training year was a categorical variable with three levels, this factor was not included in the subsequent regression and interaction analyses for the BSMAS scores to maintain model parsimony and ease of interpretation. However, to elucidate its impact on medical trainees’ social media behaviors, we examined its relationship with the number of frequently used social media platforms and BSMAS scores. Specifically, one-way analyses of variance were conducted to determine whether differences existed in both the platform numbers and BSMAS scores across the 3 training levels (first, second, and third years). Chi-square tests were used to compare the prevalence of SMA among these groups.

#### Correlation and Regression Analyses

To provide a preliminary overview of the interrelationships among all variables, a Spearman correlation analysis was conducted, and the results were visualized using a comprehensive heatmap that included demographic, personality, and mental health indicators. To identify the factors associated with SMA, a univariate regression analysis was used to assess the relationship between each independent variable (age, sex, personality traits, and social media usage) and the dependent variable (BSMAS score). Mental health outcomes (Patient Health Questionnaire-9, Generalized Anxiety Disorder-7, and Insomnia Severity Index) were excluded as independent variables to prevent multicollinearity and to ensure that the analysis focused on underlying predictors rather than co-occurring psychological symptoms. Following this, logistic regression was used to refine the model by selecting the most relevant variables affecting SMA (BSMAS ≥24). This approach used the full information of the scale while clarifying the most important diagnostic indicators. Stratified analyses were conducted by residency year (first, second, and third), repeating the logistic regression analysis within each subgroup.

#### Mediation and Moderation Analyses

Predictors of addictive behaviors were classified according to the interaction of the person-affect-cognition-execution model for addictive behaviors [20,21]. Accordingly, variables were categorized into relatively stable predisposing factors corresponding to the “person” component (including psychiatric history, physical health problems, and personality traits) and more dynamic factors (such as occupational burnout and night-shift frequency). We hypothesized that these dynamic factors mediate or moderate the association between stable predictors and the severity of SMA. To test this hypothesis, two sets of analyses were conducted: (1) mediation analyses to examine whether dynamic factors mediated the effects of stable predictors on BSMAS scores (using 5000 bootstrap samples) and (2) moderation analyses to evaluate whether dynamic factors modified the relationships between stable predictors and BSMAS scores.

## Results

### Demographics and Descriptive Statistics: Study Population Characteristics

A total of 3621 medical trainees were included in the analysis. The mean age was 25.3 (SD 2.5) years, and the sample included 1567 (43.2%) males and 2054 (56.8%) females. Participants were well distributed across residency years. Overall, 1065 (29.4%) participants reported physical health problems, 707 (19.5%) reported a psychiatric history, 472 (13%) had received prior psychological treatment, and 1176 (32.5%) reported a financial burden. Nearly 60% (n=2173) of participants were only children. Detailed demographic characteristics are presented in Table 1, and post hoc results are provided in Tables S1-S9 and Figures S1-S3 in Multimedia Appendix 1.

**Table 1.** Demographic and baseline health characteristics of Chinese medical trainees enrolled in standardized residency training programs who participated in a nationwide cross-sectional anonymous online survey conducted from August 29, 2024, to September 10, 2024 (N=3621).

Characteristics	Values
Age (years), mean (SD)	25.3 (2.5)
Sex, n (%)	
Male	1567 (43.2)
Female	2054 (56.8)
Training year, n (%)	
First year	1657 (45.7)
Second year	1159 (32)
Third year	805 (22.2)
Physical health problems (yes), n (%)	1065 (29.4)
Psychiatric history (yes), n (%)	707 (19.5)
Financial burden (yes), n (%)	1176 (32.5)
Only child status (yes), n (%)	2139 (59)
Prior psychological treatment (yes), n (%)	472 (13)

### Demographics and Work-Related Characteristics

Sex distribution differed significantly across social media usage groups (*χ*²_4_=49.786; *P*<.001). When distributions were examined within sex, a higher proportion of female medical trainees reported social media use of 2 hours or more per day (1233/2054, 60.1%) compared with male medical trainees (728/1567, 46.5%). Usage duration was significantly associated with physical health problems (*χ*²_4_=29.894; *P*<.001) and psychiatric history (*χ*²_4_=20.447; *P*<.001), with higher prevalence observed at both extremes of social media use, defined as minimal use (≤1 h/d) and excessive use (>4 h/d), compared with moderate use. Financial burden also differed significantly across usage groups (*χ*²_4_=33.250; *P*<.001). No significant differences were detected for age, prior psychological treatment, or only-child status. Regarding work-related variables, both minimal and excessive users reported higher occupational burnout scores than moderate users (*F*_4, 3616_=14.393; *P*<.001). Night-shift frequency varied across groups (*F*_4, 3616_=5.088; *P*<.001), with higher averages observed among minimal users. Training year distribution differed significantly across usage categories (*χ*²_8_=25.383; *P*=.001), with excessive social media use becoming more prevalent across advancing residency years. Details are presented in Table 2.

**Table 2.** Demographic and work-related characteristics of Chinese medical trainees, stratified by daily social media use duration (N=3621)^a^.

Characteristic	≤1 h (n=404)	1‐2 hours (n=1256)	2‐3 hours (n=1069)	3‐4 hours (n=473)	>4 hours (n=419)	Statistics	*P* value
Age (years), mean (SD)	25.5 (2.64)	25.39 (2.58)	25.19 (2.36)	25.24 (2.30)	25.34 (2.39)	1.638^b^ (4, 3616)	.16
Sex, n (%)						49.786^c^ (4)	<.001
Male	225 (14.4)	741 (47.3)	387 (24.7)	174 (11.1)	167 (10.7)		
Female	179 (8.7)	515 (25.1)	682 (33.2)	299 (14.6)	252 (12.3)		
Physical health problems, n (%)	153 (37.9)	334 (26.6)	308 (28.8)	121 (25.6)	149 (35.6)	29.894^c^ (4)	<.001
Psychiatric history, n (%)	104 (25.7)	213 (17)	193 (18.1)	88 (18.6)	97 (23.2)	20.447^c^ (4)	<.001
Prior psychological treatment, n (%)	48 (11.9)	146 (11.6)	150 (14)	69 (14.6)	59 (14.1)	5.027^c^ (4)	.29
Only child, n (%)	239 (59.2)	747 (59.5)	613 (57.3)	290 (61.3)	255 (60.9)	2.915^c^ (4)	.57
Financial burden, n (%)	179 (44.3)	412 (32.8)	309 (28.9)	143 (30.2)	133 (31.7)	33.250^c^ (4)	<.001
Training year, n (%)						25.383^c^ (8)	.001
First year	191 (11.5)	619 (37.4)	469 (28.3)	213 (12.9)	165 (10)		
Second year	114 (9.8)	386 (33.3)	376 (32.4)	144 (12.4)	139 (12)		
Third year	99 (12.3)	251 (31.2)	224 (27.8)	116 (14.4)	115 (14.3)		
Monthly night shifts, mean (SD)	6.47 (6.08)	5.44 (5.10)	5.23 (5.24)	4.98 (4.79)	5.53 (5.89)	5.088^b^ (4, 3616)	<.001
Occupational burnout, mean (SD)	2.64 (1.31)	2.28 (0.99)	2.36 (0.96)	2.21 (0.86)	2.51 (1.14)	14.393^b^ (4, 3616)	<.001

a Data were derived from a nationwide cross-sectional anonymous online survey conducted from August 29, 2024, to September 10, 2024.

b *F* test.

c Chi-square test.

### Personality Traits, Social Media Engagement, and Mental Health Outcomes

Among the Big Five personality traits, openness, conscientiousness, agreeableness, and emotional stability showed significant between-group differences (all *F*=3.09‐8.32; all *P*<.05), whereas extraversion did not. Medical trainees with moderate social media use tended to exhibit more favorable personality profiles. The number of frequently used social media platforms (*F*_4, 3616_=39.476; *P*<.001), BSMAS scores (*F*_4, 3616_=48.023; *P*<.001), and the prevalence of SMA (*χ*²_4_=43.160; *P*<.001) increased with the duration of social media use. Mental health outcomes also differed significantly by usage duration. Depression, anxiety, and insomnia scores followed a U-shaped pattern (all *F*=20.82‐24.83; all *P*<.001), with higher symptom levels observed among both minimal and excessive users compared with moderate users. Details are presented in Table 3.

**Table 3.** Personality traits, social media engagement indicators, and mental health scale scores among Chinese medical trainees, stratified by daily social media use duration (N=3621)^a^.

Characteristics	≤1 hour	1‐2 hours	2‐3 hours	3‐4 hours	>4 hours	Statistics	*P* value
Openness, mean (SD)	0.44 (2.31)	0.69 (2.21)	0.72 (2.14)	0.74 (2.21)	0.38 (2.23)	3.091^b^ (4, 3616)	.02
Conscientiousness, mean (SD)	0.65 (2.53)	1.13 (2.32)	0.86 (2.29)	0.78 (2.36)	0.54 (2.32)	6.995^b^ (4, 3616)	<.001
Extraversion, mean (SD)	–0.47 (2.66)	–0.37 (2.58)	–0.19 (2.61)	–0.42 (2.65)	–0.36 (2.71)	1.255^b^ (4, 3616)	.29
Agreeableness, mean (SD)	2.10 (2.24)	2.59 (2.04)	2.50 (2.01)	2.58 (2.06)	2.30 (2.17)	5.344^b^ (4, 3616)	<.001
Emotional stability, mean (SD)	0.82 (2.49)	1.23 (2.39)	1.04 (2.35)	1.22 (2.40)	0.52 (2.47)	8.319^b^ (4, 3616)	<.001
Number of platforms, mean (SD)	2.32 (1.66)	2.80 (1.42)	3.14 (1.43)	3.29 (1.38)	3.36 (1.70)	39.476 (4, 3616)	<.001
BSMAS^c^ score, mean (SD)	13.07 (5.57)	14.18 (4.78)	15.64 (4.69)	15.92 (4.93)	16.90 (5.36)	48.023 (4, 3616)	<.001
SMA^d^, n (%)	19 (4.7)	46 (3.7)	63 (5.9)	32 (6.8)	51 (12.2)	43.160^e^ (4)	<.001
PHQ-9^f^, mean (SD)	9.54 (8.01)	6.63 (5.78)	6.6 (5.27)	6.4 (4.96)	7.89 (6.3)	24.825 (4, 3616)	<.001
GAD-7^g^, mean (SD)	7.07 (6.56)	4.82 (4.97)	4.81 (4.54)	4.39 (4.31)	5.78 (5.49)	21.607 (4, 3616)	<.001
ISI^h^, mean (SD)	9.99 (8.02)	7.09 (5.94)	7.03 (5.81)	6.84 (5.66)	7.91 (6.68)	20.822 (4, 3616)	<.001

a Data were derived from a nationwide cross-sectional anonymous online survey conducted from August 29, 2024, to September 10, 2024.

b *F* test.

c BSMAS: Bergen Social Media Addiction Scale.

d SMA: social media addiction; defined as a BSMAS score ≥24.

e Chi-square test.

f PHQ-9: 9-item Patient Health Questionnaire.

g GAD-7: 7-item Generalized Anxiety Disorder.

h ISI: Insomnia Severity Index.

### Correlation Analysis

**Figure 1.**
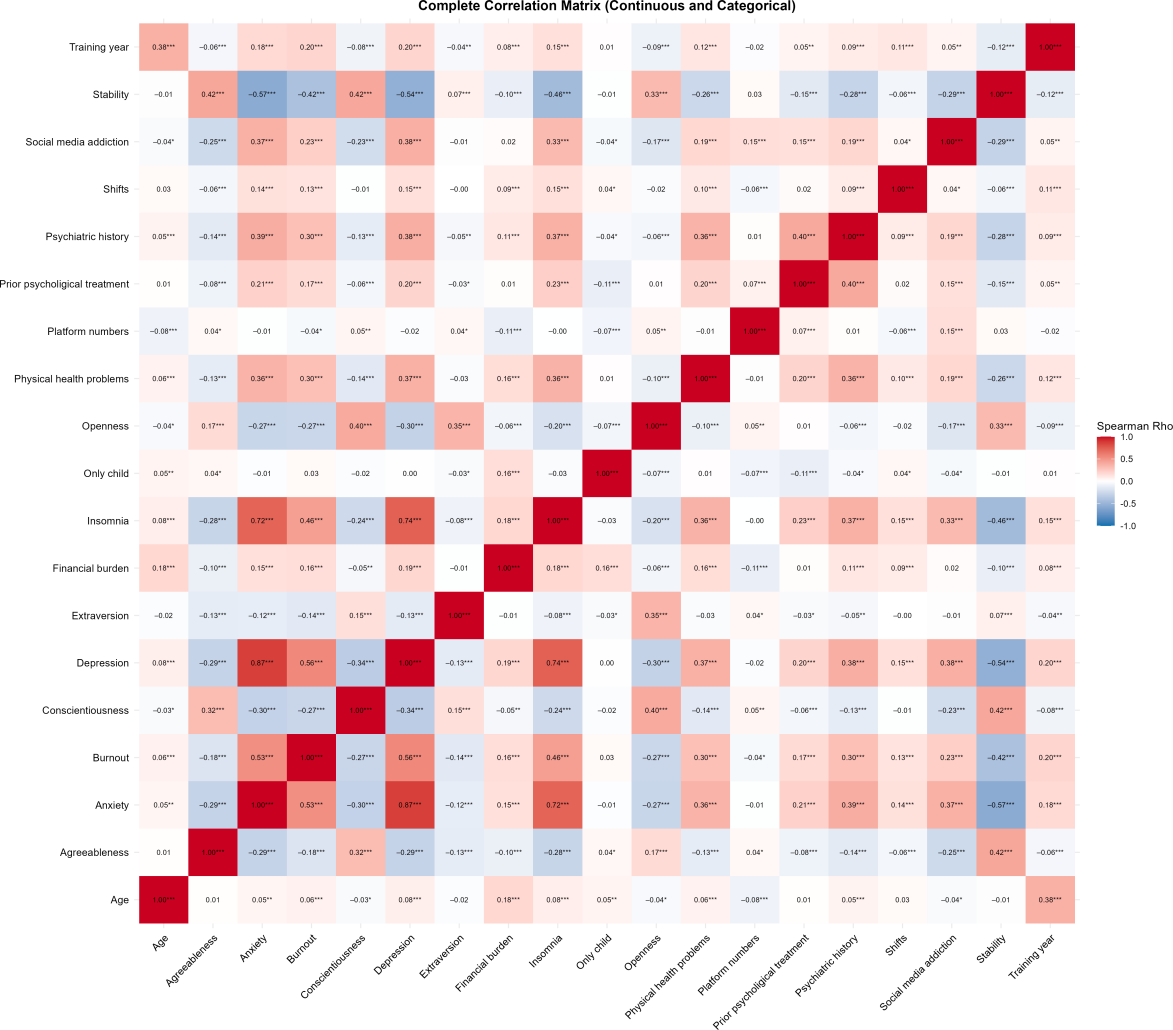
Spearman correlation heatmap of demographic, workload, personality, and mental health variables among Chinese medical trainees (N=3621). Data were derived from a nationwide cross-sectional anonymous online survey conducted from August 29, 2024, to September 10, 2024. The heatmap illustrates intercorrelations among all analyzed variables, with the color gradient representing the strength and direction of associations (red: positive; blue: negative). Numbers within cells indicate correlation coefficients. Categorical and ordinal variables were coded as follows: physical health problems, psychiatric history, prior psychological treatment, and financial burden (1=“no” and 2=“yes”); only-child status (1=“yes” and 2=“no”); and training year (1=“year 1,” 2=“year 2,” and 3=“year 3”). BSMAS: Bergen Social Media Addiction Scale; ISI: Insomnia Severity Index; SM: social media. *P<.05, **P<.01, ***P<.001.

The Spearman correlation heatmap (Figure 1) provides a comprehensive overview of the interrelationships among demographic, personality, and mental health variables. Specifically, BSMAS scores, as an index of SMA, showed significant positive correlations with occupational burnout (*r*=0.23; *P*<.001), the number of frequently used platforms (*r*=0.15; *P*<.001), and monthly night shifts (*r*=0.04; *P*=.029). Regarding mental health outcomes, BSMAS scores exhibited robust positive associations with depression (*r*=0.38; *P*<.001), anxiety (*r*=0.37; *P*<.001), and insomnia (*r*=0.33; *P*<.001). Furthermore, significant correlations were observed between BSMAS and several categorical risk factors: physical health problems (*r*=0.19; *P*<.001), psychiatric history (*r*=0.19; *P*<.001), and prior psychological treatment (*r*=0.15, *P*<.001) were all positively associated with addiction severity. In terms of personality traits, conscientiousness (*r*=–0.23; *P*<.001), stability (*r*=–0.29; *P*<.001), and agreeableness (*r*=–0.25; *P*<.001) exhibited significant negative correlations with BSMAS scores. These preliminary findings suggest that SMA is closely associated with systemic occupational stress and individual psychological vulnerabilities.

### Regression Analysis

**Figure 2.**
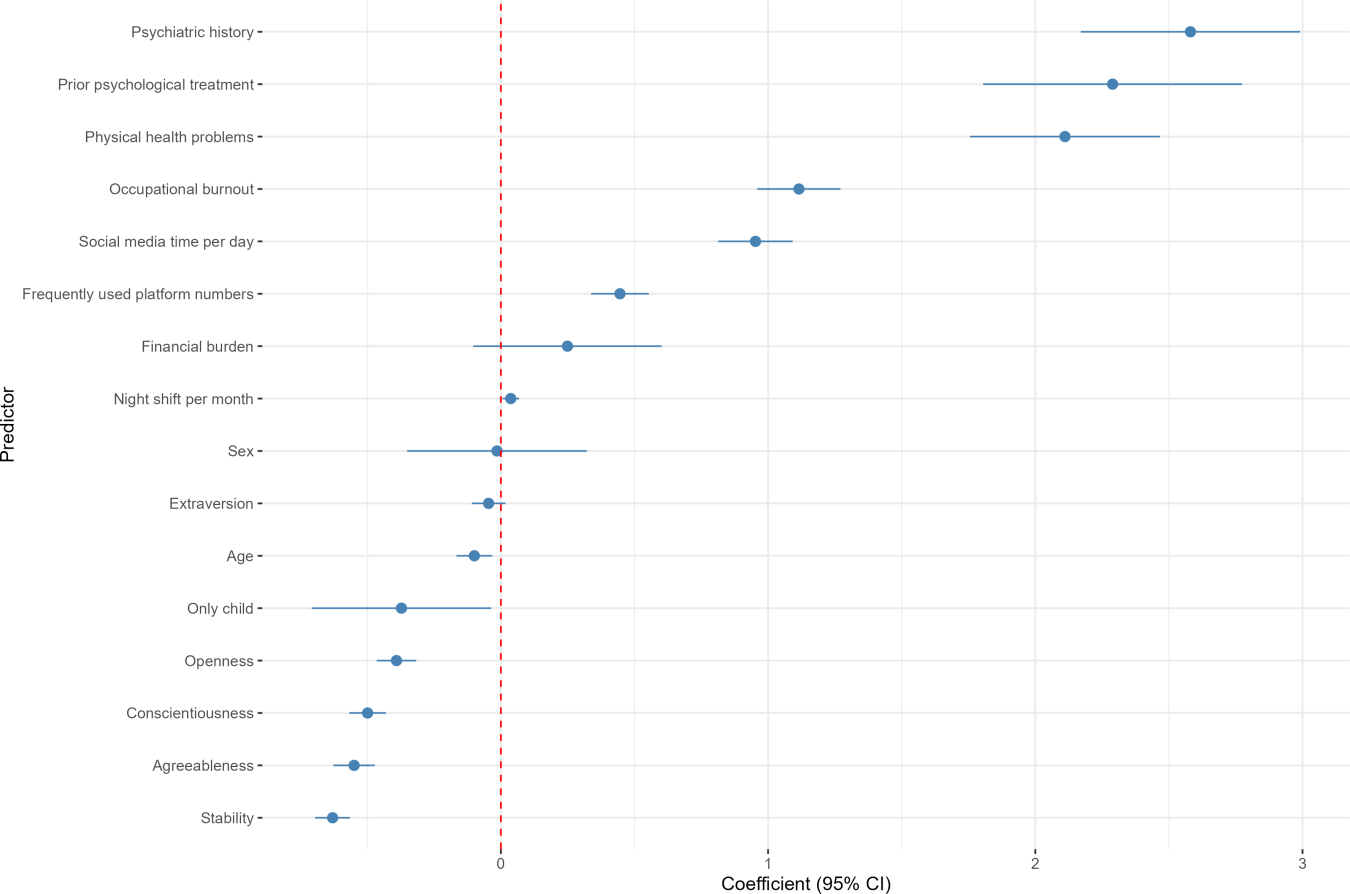
Forest plot of univariable linear regression coefficients for predictors of Bergen Social Media Addiction Scale (BSMAS) scores among Chinese medical trainees (N=3621). Data were derived from a nationwide cross-sectional anonymous online survey conducted from August 29, 2024, to September 10, 2024. Regression coefficients (solid points) and 95% CIs (horizontal lines) are shown for demographic, occupational, personality, and health-related predictors. The dashed vertical line indicates a coefficient of zero (no association). Categorical variables were coded as follows: physical health problems, psychiatric history, prior psychological treatment, and financial burden (1=“no” and 2=“yes”) and only-child status (1=“yes” and 2=“no”).

Univariate linear regression analyses identified several significant predictors of BSMAS scores (Figure 2; Table S10 in Multimedia Appendix 1). Higher occupational burnout, more frequent night shifts, longer daily social media use, female sex, physical health problems, and psychiatric history were positively associated with BSMAS scores (β=.15 to .35; all *P*<.05). In contrast, conscientiousness was inversely associated with BSMAS scores (β=–.22; *P*=.003). No other variables were significantly associated with BSMAS scores.

**Figure 3.**
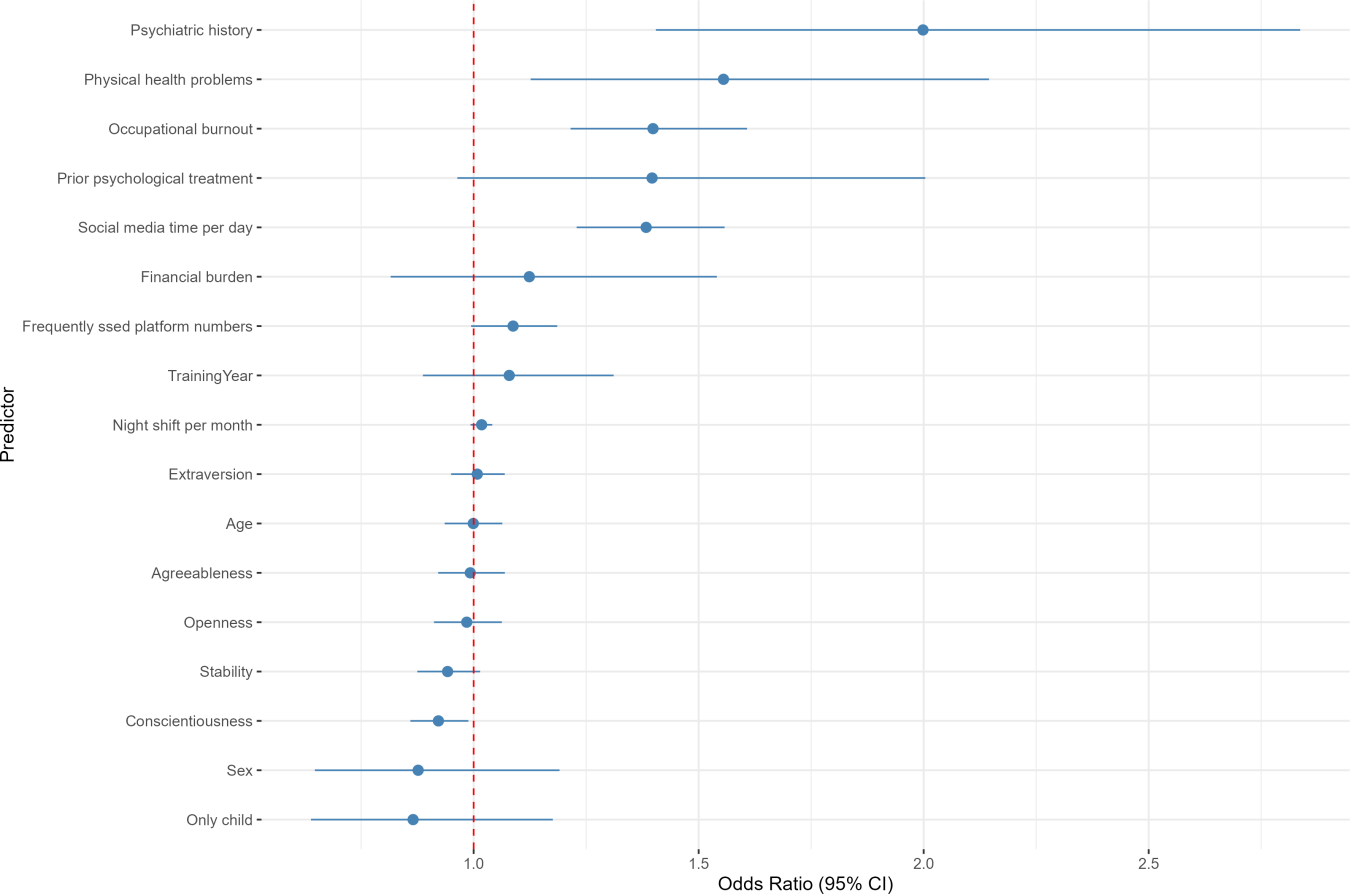
Forest plot of multivariable logistic regression predictors of probable social media addiction (Bergen Social Media Addiction Scale [BSMAS] ≥24) among Chinese medical trainees (N=3621). Data were derived from a nationwide cross-sectional anonymous online survey conducted from August 29, 2024, to September 10, 2024. Odds ratios (ORs; solid points) and 95% CIs (horizontal lines) are displayed for each predictor. The dashed vertical line denotes a null effect (OR 1.00). Categorical variables were coded as follows: physical health problems, psychiatric history, prior psychological treatment, and financial burden (1=“no” and 2=“yes”) and only-child status (1=“yes” and 2=“no”).

When using a BSMAS score of 24 or more to define SMA, only a subset of predictors remained significant in the logistic regression model (Figure 3; Table S11 in Multimedia Appendix 1). Specifically, higher occupational burnout was significantly associated with increased odds of developing SMA (odds ratio [OR] 1.409, 95% CI 1.226‐1.617; *P*<.001). Similarly, longer daily social media use (OR 1.388, 95% CI 1.233‐1.562; *P*<.001), physical health problems (OR 1.564, 95% CI 1.133‐2.157; *P*=.006), and a positive psychiatric history (OR 2.000, 95% CI 1.406‐2.838; *P*<.001) were all associated with an elevated risk of addiction. In contrast, higher conscientiousness showed a protective effect (OR 0.922, 95% CI 0.860‐0.989; *P*=.02).

### Mediation Analysis

Occupational burnout was positively correlated with both psychiatric history (β=.809; *P*<.001) and physical health problems (β=.681; *P*<.001) and was negatively associated with conscientiousness (β=–.119; *P*<.001). Mediation analysis revealed that burnout significantly mediated the effects of psychiatric history and physical health problems on BSMAS scores, with mediation proportions of 28.1% (indirect effect β=.726; bootstrap standard error [BootSE]=0.090; *P*<.001) and 29.6% (indirect effect β=.626; BootSE=0.074; *P*<.001), respectively. Furthermore, the protective effect of conscientiousness on BSMAS scores was partially mediated by burnout, contributing a negative mediation effect with a proportion of 20.9% (indirect effect β=–.104; BootSE=0.013; *P*<.001).

**Figure 4.**
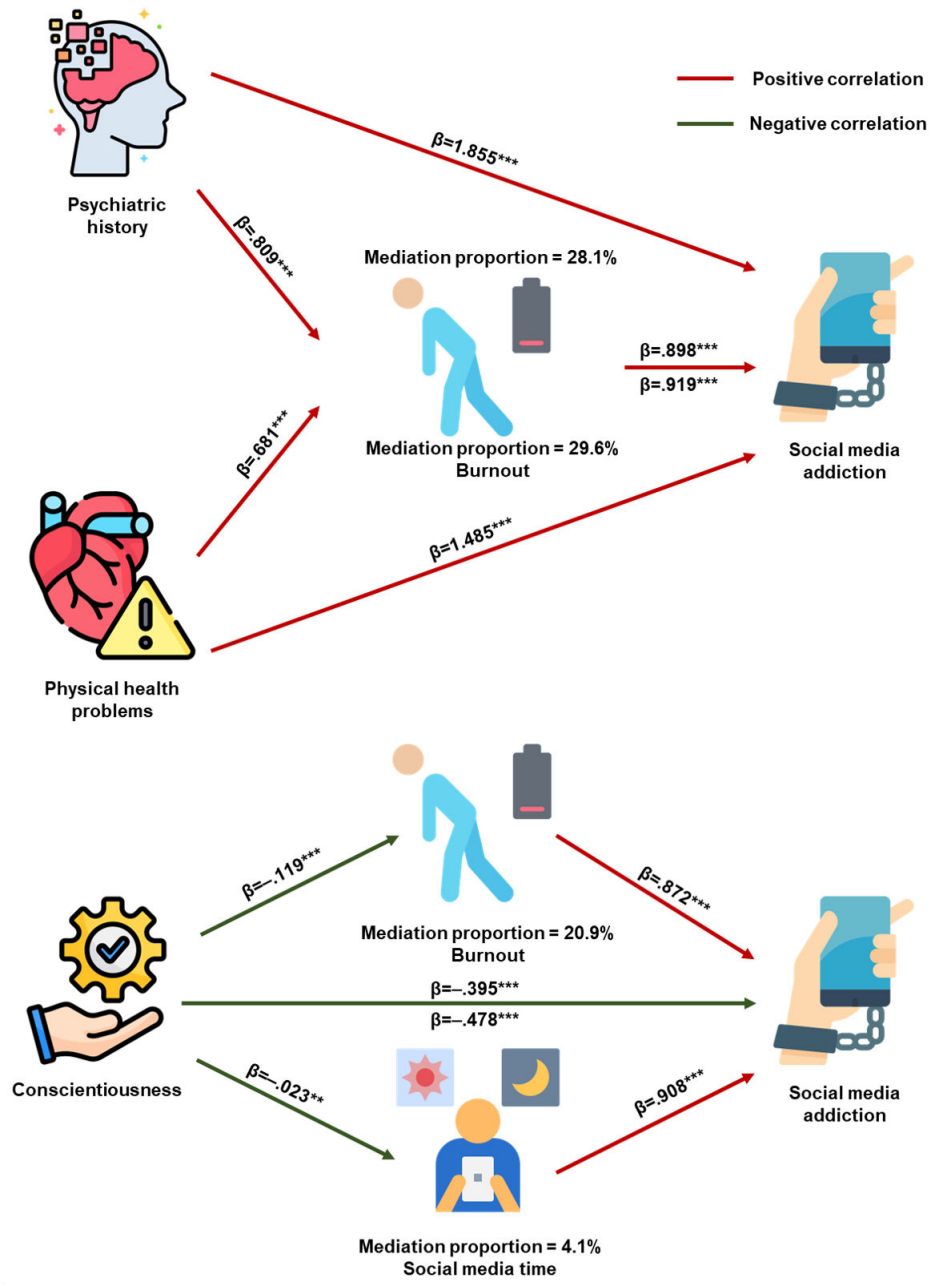
Parallel mediation models evaluating indirect associations between health/personality factors and social media addiction severity via occupational burnout and daily social media use duration among Chinese medical trainees (N=3621). Data were derived from a nationwide cross-sectional anonymous online survey conducted from August 29, 2024, to September 10, 2024. The models illustrate how psychiatric history, physical health problems, and conscientiousness are associated with Bergen Social Media Addiction Scale scores through two mediators: (1) occupational burnout and (2) daily social media use duration. Values on the paths represent standardized regression coefficients, with significance levels denoted by *P<.05, **P<.01, ***P<.001.

In contrast, daily social media use time was not significantly associated with psychiatric history or physical health problems but was negatively correlated with conscientiousness (β=–.023; *P*=.008). Mediation analysis indicated that daily social media use time modestly mediated the association between conscientiousness and BSMAS scores, with a mediation proportion of 4.1% (indirect effect β=–.021; BootSE=0.008; *P*=.009). For more detailed results, refer to Figure 4 and Table S12 in Multimedia Appendix 1.

### Moderation Analysis

Moderation analyses revealed significant interaction effects in both models (Table S13 and Figure S4 in Multimedia Appendix 1). Burnout significantly moderated the association between psychiatric history and BSMAS scores (β=−.795; *P*<.001), such that the effect of psychiatric history weakened at higher levels of burnout; correspondingly, the proportion of SMA increased across burnout levels among individuals with a psychiatric history (eg, 14.4%, 10.3%, 15%, 17%, and 19.6%). A similar moderating pattern was observed for physical health problems (β=−.513; *P*=.002), with SMA prevalence likewise increasing across burnout levels among affected individuals (eg, 8.5%, 7.8%, 11.4%, 14%, and 16.1%). No other interaction effects were statistically significant.

### Stratified Analysis

In stratified analyses by training year, occupational burnout consistently predicted higher odds of SMA across all subgroups (year 1: OR 1.327, 95% CI 1.112‐1.582, *P*=.002; year 2: OR 1.442, 95% CI 1.201‐1.729, *P*<.001; year 3: OR 1.391, 95% CI 1.123‐1.722, *P*=.002), fully aligning with the overall sample. Physical health problems were associated with addiction only in year 2 medical trainees (OR 1.675, 95% CI 1.154‐2.429; *P*=.007). Psychiatric history showed significant effects in both year 2 (OR 1.675, 95% CI 1.154‐2.429; *P*=.007) and year 3 medical trainees (OR 1.586, 95% CI 1.039‐2.420; *P*=.03) but not in year 1 medical trainees. Daily social media use time was not significant in the stratified models (all *P*>.100). Conscientiousness was protective only in year 1 (OR 0.918, 95% CI 0.850‐0.991; *P*=.03), while showing null associations in years 2 and 3. Beyond these 5 primary predictors, night-shift frequency was an additional significant factor in year 2 medical trainees (OR 1.041, 95% CI 1.008‐1.076; *P*=.02), suggesting a unique role of workload intensity at this training stage. For more detailed results, refer to Tables S14-S16 in Multimedia Appendix 1.

## Discussion

### Principal Findings

In this large cross-sectional survey of 3621 medical trainees in China, we found that the prevalence of SMA (BSMAS ≥24) was 5.8% (211/3621), with the highest proportion among second-year medical trainees (92/1159, 7.9%) and among those using social media for more than 4 hours per day (12.2%). Higher burnout, longer daily use, physical health problems, and psychiatric history significantly increased the odds of developing SMA, whereas conscientiousness had a protective effect. In addition, we observed U-shaped associations between daily social media use and occupational burnout, physical health problems, psychiatric history, and mental health outcomes (depression, anxiety, and insomnia), all of which were linked to less favorable outcomes than moderate use. Exploratory analyses further suggested that burnout and time spent on social media functioned as key pathways linking health vulnerabilities and personality traits to SMA.

### SMA Prevalence and Its Relationship With Mental Health Outcomes

Meta-analytic evidence using the BSMAS with a clinical cutoff score of 24 indicates a pooled global prevalence of SMA of approximately 8% (95% CI 4%‐12%) [18]. In this study, the prevalence of SMA among medical trainees was 5.8%. Although numerically lower than the pooled global estimate, this rate remains clinically concerning given the professional demands and vulnerability of this population. Notably, this prevalence was substantially higher than the 3.49% reported among Chinese adolescents when identical diagnostic criteria were applied [17]. Furthermore, we observed that the prevalence of SMA doubled or even tripled as daily usage transitioned from moderate to excessive levels (3.7%‐6.8% for ≤4 h vs 12.2% for >4 h). This nonlinear escalation suggests that excessive social media use represents a critical tipping point at which behavioral engagement shifts from routine activity to clinically significant addiction. Importantly, irrespective of the threshold or classification approach, SMA has been consistently linked to adverse mental health outcomes. Recent longitudinal studies and meta-analyses have demonstrated robust associations between SMA and impaired concentration, chronic sleep disturbances (particularly insomnia), and a markedly increased risk of depression and suicidal ideation [22,23]. Our findings further corroborate these associations, providing additional evidence that SMA is closely related to poor mental health outcomes among medical trainees.

### U-Shaped Associations Between Social Media Use and Mental Health Outcomes

Social media usage has long been considered a potential correlate of mental health problems [3]. Based on large-scale survey data from Chinese medical trainees, both excessive and minimal social media use were closely associated with higher levels of burnout, depression, anxiety, and insomnia. Notably, the ≤1-hour usage group reported greater financial burden and more frequent night shifts, suggesting substantial occupational constraints. These U-shaped patterns may reflect 2 behavioral phenotypes under occupational stress: minimal use associated with withdrawal or disengagement, and excessive use potentially reflecting emotion-focused or avoidant coping in response to distress. Both patterns were linked to poorer mental health outcomes than moderate use, warranting longitudinal validation. These findings argue against a simple linear relationship between the duration of social media use and psychological and physical health outcomes.

### Occupational Burnout as a Pathway and Amplifier in SMA

In both the overall sample and the stratified analyses, occupational burnout consistently emerged as a significant predictor of SMA. Recognizing burnout as a modifiable risk factor creates opportunities for targeted interventions in medical training environments [24,25]. Furthermore, our findings indicate that burnout functions as a mediating factor, amplifying the adverse effects of psychiatric history and physical health problems on SMA while attenuating the protective role of conscientiousness. This dual role underscores its centrality as a target for interventions aimed at reducing digital overreliance and safeguarding the mental health of medical trainees [26].

The association between burnout and SMA may be driven by a dysregulated neurobiological stress response involving the hypothalamic-pituitary-adrenal axis and the mesocorticolimbic reward system [27]. Chronic stress related to burnout can create prolonged hypothalamic-pituitary-adrenal axis hyperactivity, eventually leading to neuroendocrine exhaustion and an “antireward” brain state [28]. In this state, natural rewards (eg, clinical success) become less satisfying, and the brain becomes sensitized to dopamine surges from digital microrewards such as “likes” and notifications. Consequently, burned-out medical trainees may engage in compulsive social media use as a maladaptive form of “self-medication” to counteract anhedonia and emotional numbness, creating a pathological cycle of addiction [29].

### Interpretation of Stratified Findings

Stratified analyses confirmed that burnout remained a consistent predictor of SMA across all residency years. In contrast, other predictors showed stage-specific effects. Physical health problems and psychiatric history were significant only in years 2 and 3, suggesting cumulative vulnerability to prolonged stress exposure. Furthermore, personality traits played a pivotal role in mental health [30]; specifically, conscientiousness was protective only in year 1, indicating an early buffering effect. Night-shift frequency uniquely predicted addiction in year 2, highlighting this stage as a critical inflection point at which workload and circadian disruption may increase risk.

### Comparison With Prior Studies and Cross-Cultural Perspectives

Our findings are broadly consistent with and extend evidence from other cultural settings. For example, studies in Saudi Arabia have linked SMA among medical students to higher anxiety, poorer academic performance, and greater burnout [31,32]. Tunisian data indicate associations with reduced self-esteem [33], whereas Ugandan data show strong links between problematic digital use and depression [34]. Multicountry research has further confirmed that increased social media use is associated with psychological distress and impaired academic outcomes through maladaptive coping [35]. Collectively, these studies suggest that the interplay between social media use, addiction risk, and burnout transcends cultural boundaries, reinforcing burnout as a universal intervention target while underscoring the influence of contextual factors, such as training demands and social support.

### Limitations

First, the cross-sectional design prevents the establishment of definitive cause-and-effect relationships. Although our findings suggest that burnout increases the risk of SMA development, a bidirectional relationship is also possible. Future research should use longitudinal designs to determine causality more precisely. Second, the data were based on self-reports, which are susceptible to recall and social desirability biases and may have affected the accuracy of the prevalence and usage findings. Future studies could enhance data accuracy by incorporating objective measures of social media use. Third, the focus on Chinese medical trainees restricts the generalizability of the findings to other populations. Social and cultural backgrounds, health care systems, and social media usage habits may vary significantly across countries. Future studies should conduct cross-cultural comparisons to validate these findings.

### Future Directions

To build on the foundation of this study, future research should adopt longitudinal designs to establish causal relationships among occupational burnout, physical health problems, and the development of SMA. Qualitative research could provide valuable context by exploring the lived experiences of both minimal and excessive social media users, helping to validate and enrich the “2 phenotypes” hypothesis. Intervention studies are needed to test the efficacy of burnout-focused programs in reducing the prevalence and severity of SMA in this population. These studies would provide evidence of effective strategies for addressing this emerging health issue.

### Conclusions

This study provides a systematic characterization of SMA among Chinese medical trainees. Occupational burnout emerged as the most consistent correlate of SMA, operating both as an independent risk factor and as a pathway that amplifies the effects of psychiatric history, physical health problems, and personality traits. A U-shaped association was identified between the duration of social media use and adverse mental health outcomes, with both minimal and excessive use associated with less favorable profiles than moderate use, suggesting distinct but maladaptive patterns of engagement under occupational stress. Stratified analyses further indicated stage-specific vulnerabilities across residency years. Together, these findings suggest that SMA in medical trainees reflects the interaction among systemic workload stressors, individual vulnerability, and behavioral coping patterns. Accordingly, interventions should move beyond monitoring screen time and prioritize burnout reduction and supportive training environments to mitigate addiction risk.

## Supplementary material

10.2196/75675 Multimedia Appendix 1 Additional documentation and detailed statistical evidence supporting the study findings.

10.2196/75675 Checklist 1 CHERRIES checklist.

## Abbreviations

BootSE: bootstrap standard error
BSMAS: Bergen Social Media Addiction Scale
CHERRIES: Checklist for Reporting Results of Internet E-Surveys
OR: odds ratio
SMA: social media addiction
SRT: standardized residency training
