# Supplementary material for: Prevalence of Social Media Addiction and Associations With Usage Patterns, Burnout, and Health Conditions Among Medical Trainees in China: Cross-Sectional Study
Source: J Med Internet Res. 2026 May 4;28:e75675. doi: 10.2196/75675 (PMC13138709; doi:10.2196/75675)
Supplement: Multimedia Appendix 1 [file jmir-v28-e75675-s001.docx]

## Supplement Materials

## Questionnaires

The original questions used in this study are provided below; standardized questionnaires are not listed. These translated questions are listed to ensure that other researchers can accurately replicate the study, thereby validating and expanding upon the findings.

**1. Gender: What is your gender?**

Male

Female

**2. Age: Please enter your age (in years).**

**3. Training Year: What year of residency training are you currently in?**

First year

Second year

Third year

4**. Social Media Usage Time: How many hours do you spend on social media per day?**

○N ≤ 1

○1＜N ≤ 2

○2＜N ≤ 3

○3＜N ≤ 4

○4＜N ≤ 5

○N＞5

**5. Physical Health Problems: Do you have any chronic physical health issues that require long-term attention?**

No

Yes

**6. Psychiatric History: Have you ever been diagnosed with mental health issues such as anxiety or depression?**

No

Yes

**7. Psychological Treatment: Have you ever received psychological counseling or therapy?**

No

Yes

**8. Night Shifts: How many night shifts do you work on average per month?**

**9. Occupational Burnout: What is your current level of occupational burnout?**

○1 I enjoy my work and have no symptoms of occupational burnout

○2 I occasionally feel stressed and less energetic, but I don’t consider myself burned out

○3 I definitely feel burned out and have one or more symptoms, such as physical and emotional exhaustion

○4 My burnout symptoms are persistent, and I often think about frustrations at work

○5 I am completely burned out, often doubting whether I can continue. I may need to make some changes or seek help

**10. Financial Burden: Do you have any family financial burdens (e.g., providing financial support to parents or other family members, or supporting children)?**

No

Yes

**11. Single Child: Are you an only child?**

Yes

No

# Stratified Analyses by Residency Year

Stratified logistic regression analyses were conducted by residency year. The results demonstrated that occupational burnout was a consistent risk factor across all subgroups, while several predictors exhibited year-specific effects.

First-year residents (n = 1,657): Occupational burnout significantly increased the odds of social media addiction (OR = 1.327, 95% CI [1.112–1.582], p = 0.002). Conscientiousness showed a significant protective effect (OR = 0.918, 95% CI [0.850–0.991], p = 0.028). Openness demonstrated a marginally protective trend (OR = 0.935, 95% CI [0.867–1.008], p = 0.089), though not reaching conventional significance. Other predictors, including age, sex, number of night shifts, and psychiatric history, were not significant (all p > 0.100).

Second-year residents (n = 1,159): Occupational burnout was again a robust predictor (OR = 1.442, 95% CI [1.201–1.729], p < 0.001). The number of monthly night shifts significantly increased addiction risk (OR = 1.041, 95% CI [1.008–1.076], p = 0.015). Psychiatric history was also a significant risk factor (OR = 1.675, 95% CI [1.154–2.429], p = 0.007). None of the Big Five personality traits showed significant associations (all p > 0.100).

Third-year residents (n = 805): Burnout again emerged as a strong risk factor (OR = 1.391, 95% CI [1.123–1.722], p = 0.002). Psychiatric history was also significantly associated with increased risk of social media addiction (OR = 1.586, 95% CI [1.039–2.420], p = 0.033). In this subgroup, neither conscientiousness (OR = 0.968, 95% CI [0.871–1.076], p = 0.546) nor openness (OR = 1.027, 95% CI [0.929–1.136], p = 0.613) were significant predictors. The number of night shifts showed no association (OR = 1.012, 95% CI [0.981–1.044], p = 0.430).

Summary: Across all training stages, occupational burnout consistently predicted higher odds of social media addiction, with ORs ranging from 1.327 to 1.442. Conscientiousness was protective only in first-year residents (OR = 0.918, p = 0.028), while openness showed a marginal trend in this group (OR = 0.935, p = 0.089). The effect of night shifts was specific to second-year residents (OR = 1.041, p = 0.015), highlighting the burden of clinical workload during this phase. Psychiatric history was a significant risk factor in second- and third-year residents (OR = 1.675, p = 0.007; OR = 1.586, p = 0.033, respectively), but not in first-year residents.

# Multicollinearity Assessment

To assess multicollinearity, we conducted a linear regression using the same set of independent variables as in our logistic regression model (with BSMAS score as the dependent variable). This approach is commonly applied since VIF statistics are not directly available for logistic regression. The results showed that all variance inflation factors (VIFs) were well below the conventional thresholds (all < 2), indicating no evidence of problematic multicollinearity among the predictors (see Supplementary Table S17).

## Supplement Table S1 LSD Post Hoc Analysis Results for Various Variables Among Social Media Time Groups

| **Dependent Variable** | **(I) Social Media Time Group** | **(J) Social Media Time Group** | **Mean Difference (I-J)** | **Std. Error** | **Sig.** | **95% Confidence Interval** | |
| --- | --- | --- | --- | --- | --- | --- | --- |
|  |  |  |  |  |  | **Lower Bound** | **Upper Bound** |
| Age | 1 | 2 | .107 | .141 | .450 | -.17 | .38 |
|  |  | 3 | .307* | .144 | .033 | .02 | .59 |
|  |  | 4 | .254 | .167 | .128 | -.07 | .58 |
|  |  | 5 | .154 | .172 | .371 | -.18 | .49 |
|  | 2 | 1 | -.107 | .141 | .450 | -.38 | .17 |
|  |  | 3 | .200 | .103 | .051 | .00 | .40 |
|  |  | 4 | .148 | .133 | .267 | -.11 | .41 |
|  |  | 5 | .047 | .139 | .734 | -.23 | .32 |
|  | 3 | 1 | -.307* | .144 | .033 | -.59 | -.02 |
|  |  | 2 | -.200 | .103 | .051 | -.40 | .00 |
|  |  | 4 | -.052 | .136 | .701 | -.32 | .21 |
|  |  | 5 | -.153 | .142 | .283 | -.43 | .13 |
|  | 4 | 1 | -.254 | .167 | .128 | -.58 | .07 |
|  |  | 2 | -.148 | .133 | .267 | -.41 | .11 |
|  |  | 3 | .052 | .136 | .701 | -.21 | .32 |
|  |  | 5 | -.101 | .166 | .544 | -.43 | .22 |
|  | 5 | 1 | -.154 | .172 | .371 | -.49 | .18 |
|  |  | 2 | -.047 | .139 | .734 | -.32 | .23 |
|  |  | 3 | .153 | .142 | .283 | -.13 | .43 |
|  |  | 4 | .101 | .166 | .544 | -.22 | .43 |
| Shifts per Month | 1 | 2 | 1.028* | .304 | .001 | .43 | 1.62 |
|  |  | 3 | 1.231* | .310 | .000 | .62 | 1.84 |
|  |  | 4 | 1.489* | .360 | .000 | .78 | 2.19 |
|  |  | 5 | .938* | .371 | .011 | .21 | 1.66 |
|  | 2 | 1 | -1.028* | .304 | .001 | -1.62 | -.43 |
|  |  | 3 | .203 | .221 | .358 | -.23 | .64 |
|  |  | 4 | .460 | .287 | .108 | -.10 | 1.02 |
|  |  | 5 | -.090 | .300 | .763 | -.68 | .50 |
|  | 3 | 1 | -1.231* | .310 | .000 | -1.84 | -.62 |
|  |  | 2 | -.203 | .221 | .358 | -.64 | .23 |
|  |  | 4 | .257 | .293 | .381 | -.32 | .83 |
|  |  | 5 | -.294 | .306 | .338 | -.89 | .31 |
|  | 4 | 1 | -1.489* | .360 | .000 | -2.19 | -.78 |
|  |  | 2 | -.460 | .287 | .108 | -1.02 | .10 |
|  |  | 3 | -.257 | .293 | .381 | -.83 | .32 |
|  |  | 5 | -.551 | .357 | .123 | -1.25 | .15 |
|  | 5 | 1 | -.938* | .371 | .011 | -1.66 | -.21 |
|  |  | 2 | .090 | .300 | .763 | -.50 | .68 |
|  |  | 3 | .294 | .306 | .338 | -.31 | .89 |
|  |  | 4 | .551 | .357 | .123 | -.15 | 1.25 |
| Burnout | 1 | 2 | .364* | .059 | .000 | .25 | .48 |
|  |  | 3 | .287* | .060 | .000 | .17 | .40 |
|  |  | 4 | .436* | .070 | .000 | .30 | .57 |
|  |  | 5 | .138 | .072 | .055 | .00 | .28 |
|  | 2 | 1 | -.364* | .059 | .000 | -.48 | -.25 |
|  |  | 3 | -.077 | .043 | .072 | -.16 | .01 |
|  |  | 4 | .072 | .055 | .192 | -.04 | .18 |
|  |  | 5 | -.227* | .058 | .000 | -.34 | -.11 |
|  | 3 | 1 | -.287* | .060 | .000 | -.40 | -.17 |
|  |  | 2 | .077 | .043 | .072 | -.01 | .16 |
|  |  | 4 | .149* | .057 | .008 | .04 | .26 |
|  |  | 5 | -.150* | .059 | .011 | -.27 | -.03 |
|  | 4 | 1 | -.436* | .070 | .000 | -.57 | -.30 |
|  |  | 2 | -.072 | .055 | .192 | -.18 | .04 |
|  |  | 3 | -.149* | .057 | .008 | -.26 | -.04 |
|  |  | 5 | -.299* | .069 | .000 | -.43 | -.16 |
|  | 5 | 1 | -.138 | .072 | .055 | -.28 | .00 |
|  |  | 2 | .227* | .058 | .000 | .11 | .34 |
|  |  | 3 | .150* | .059 | .011 | .03 | .27 |
|  |  | 4 | .299* | .069 | .000 | .16 | .43 |
| Openness | 1 | 2 | -.251* | .126 | .046 | -.50 | .00 |
|  |  | 3 | -.284* | .129 | .027 | -.54 | -.03 |
|  |  | 4 | -.298* | .149 | .046 | -.59 | .00 |
|  |  | 5 | .061 | .154 | .691 | -.24 | .36 |
|  | 2 | 1 | .251* | .126 | .046 | .00 | .50 |
|  |  | 3 | -.033 | .092 | .722 | -.21 | .15 |
|  |  | 4 | -.046 | .119 | .697 | -.28 | .19 |
|  |  | 5 | .312* | .124 | .012 | .07 | .56 |
|  | 3 | 1 | .284* | .129 | .027 | .03 | .54 |
|  |  | 2 | .033 | .092 | .722 | -.15 | .21 |
|  |  | 4 | -.014 | .122 | .911 | -.25 | .23 |
|  |  | 5 | .345* | .127 | .007 | .10 | .59 |
|  | 4 | 1 | .298* | .149 | .046 | .00 | .59 |
|  |  | 2 | .046 | .119 | .697 | -.19 | .28 |
|  |  | 3 | .014 | .122 | .911 | -.23 | .25 |
|  |  | 5 | .359* | .148 | .015 | .07 | .65 |
|  | 5 | 1 | -.061 | .154 | .691 | -.36 | .24 |
|  |  | 2 | -.312* | .124 | .012 | -.56 | -.07 |
|  |  | 3 | -.345* | .127 | .007 | -.59 | -.10 |
|  |  | 4 | -.359* | .148 | .015 | -.65 | -.07 |
| Conscientiousness | 1 | 2 | -.482* | .134 | .000 | -.74 | -.22 |
|  |  | 3 | -.211 | .137 | .123 | -.48 | .06 |
|  |  | 4 | -.134 | .159 | .398 | -.45 | .18 |
|  |  | 5 | .107 | .163 | .514 | -.21 | .43 |
|  | 2 | 1 | .482* | .134 | .000 | .22 | .74 |
|  |  | 3 | .271* | .097 | .005 | .08 | .46 |
|  |  | 4 | .348* | .126 | .006 | .10 | .60 |
|  |  | 5 | .589* | .132 | .000 | .33 | .85 |
|  | 3 | 1 | .211 | .137 | .123 | -.06 | .48 |
|  |  | 2 | -.271* | .097 | .005 | -.46 | -.08 |
|  |  | 4 | .077 | .129 | .553 | -.18 | .33 |
|  |  | 5 | .317* | .135 | .019 | .05 | .58 |
|  | 4 | 1 | .134 | .159 | .398 | -.18 | .45 |
|  |  | 2 | -.348* | .126 | .006 | -.60 | -.10 |
|  |  | 3 | -.077 | .129 | .553 | -.33 | .18 |
|  |  | 5 | .241 | .157 | .125 | -.07 | .55 |
|  | 5 | 1 | -.107 | .163 | .514 | -.43 | .21 |
|  |  | 2 | -.589* | .132 | .000 | -.85 | -.33 |
|  |  | 3 | -.317* | .135 | .019 | -.58 | -.05 |
|  |  | 4 | -.241 | .157 | .125 | -.55 | .07 |
| Extraversion | 1 | 2 | -.103 | .150 | .491 | -.40 | .19 |
|  |  | 3 | -.279 | .153 | .069 | -.58 | .02 |
|  |  | 4 | -.047 | .178 | .789 | -.40 | .30 |
|  |  | 5 | -.110 | .183 | .548 | -.47 | .25 |
|  | 2 | 1 | .103 | .150 | .491 | -.19 | .40 |
|  |  | 3 | -.175 | .109 | .108 | -.39 | .04 |
|  |  | 4 | .056 | .141 | .693 | -.22 | .33 |
|  |  | 5 | -.007 | .148 | .964 | -.30 | .28 |
|  | 3 | 1 | .279 | .153 | .069 | -.02 | .58 |
|  |  | 2 | .175 | .109 | .108 | -.04 | .39 |
|  |  | 4 | .231 | .145 | .111 | -.05 | .52 |
|  |  | 5 | .169 | .151 | .265 | -.13 | .46 |
|  | 4 | 1 | .047 | .178 | .789 | -.30 | .40 |
|  |  | 2 | -.056 | .141 | .693 | -.33 | .22 |
|  |  | 3 | -.231 | .145 | .111 | -.52 | .05 |
|  |  | 5 | -.062 | .176 | .723 | -.41 | .28 |
|  | 5 | 1 | .110 | .183 | .548 | -.25 | .47 |
|  |  | 2 | .007 | .148 | .964 | -.28 | .30 |
|  |  | 3 | -.169 | .151 | .265 | -.46 | .13 |
|  |  | 4 | .062 | .176 | .723 | -.28 | .41 |
| Agreeableness | 1 | 2 | -.488* | .119 | .000 | -.72 | -.26 |
|  |  | 3 | -.397* | .121 | .001 | -.63 | -.16 |
|  |  | 4 | -.477* | .140 | .001 | -.75 | -.20 |
|  |  | 5 | -.194 | .145 | .179 | -.48 | .09 |
|  | 2 | 1 | .488* | .119 | .000 | .26 | .72 |
|  |  | 3 | .091 | .086 | .291 | -.08 | .26 |
|  |  | 4 | .010 | .112 | .928 | -.21 | .23 |
|  |  | 5 | .293* | .117 | .012 | .06 | .52 |
|  | 3 | 1 | .397* | .121 | .001 | .16 | .63 |
|  |  | 2 | -.091 | .086 | .291 | -.26 | .08 |
|  |  | 4 | -.081 | .114 | .480 | -.31 | .14 |
|  |  | 5 | .202 | .119 | .091 | -.03 | .44 |
|  | 4 | 1 | .477* | .140 | .001 | .20 | .75 |
|  |  | 2 | -.010 | .112 | .928 | -.23 | .21 |
|  |  | 3 | .081 | .114 | .480 | -.14 | .31 |
|  |  | 5 | .283* | .139 | .042 | .01 | .56 |
|  | 5 | 1 | .194 | .145 | .179 | -.09 | .48 |
|  |  | 2 | -.293* | .117 | .012 | -.52 | -.06 |
|  |  | 3 | -.202 | .119 | .091 | -.44 | .03 |
|  |  | 4 | -.283* | .139 | .042 | -.56 | -.01 |
| Stability | 1 | 2 | -.406* | .137 | .003 | -.68 | -.14 |
|  |  | 3 | -.221 | .140 | .115 | -.50 | .05 |
|  |  | 4 | -.398* | .163 | .014 | -.72 | -.08 |
|  |  | 5 | .301 | .167 | .072 | -.03 | .63 |
|  | 2 | 1 | .406* | .137 | .003 | .14 | .68 |
|  |  | 3 | .185 | .100 | .064 | -.01 | .38 |
|  |  | 4 | .008 | .130 | .953 | -.25 | .26 |
|  |  | 5 | .707* | .135 | .000 | .44 | .97 |
|  | 3 | 1 | .221 | .140 | .115 | -.05 | .50 |
|  |  | 2 | -.185 | .100 | .064 | -.38 | .01 |
|  |  | 4 | -.178 | .133 | .181 | -.44 | .08 |
|  |  | 5 | .522* | .138 | .000 | .25 | .79 |
|  | 4 | 1 | .398* | .163 | .014 | .08 | .72 |
|  |  | 2 | -.008 | .130 | .953 | -.26 | .25 |
|  |  | 3 | .178 | .133 | .181 | -.08 | .44 |
|  |  | 5 | .700* | .161 | .000 | .38 | 1.02 |
|  | 5 | 1 | -.301 | .167 | .072 | -.63 | .03 |
|  |  | 2 | -.707* | .135 | .000 | -.97 | -.44 |
|  |  | 3 | -.522* | .138 | .000 | -.79 | -.25 |
|  |  | 4 | -.700* | .161 | .000 | -1.02 | -.38 |
| Frequently  Used  Platform  Numbers | 1 | 2 | -.481* | .085 | .000 | -.65 | -.31 |
|  |  | 3 | -.813* | .087 | .000 | -.98 | -.64 |
|  |  | 4 | -.970* | .100 | .000 | -1.17 | -.77 |
|  |  | 5 | -1.039* | .103 | .000 | -1.24 | -.84 |
|  | 2 | 1 | .481* | .085 | .000 | .31 | .65 |
|  |  | 3 | -.333* | .062 | .000 | -.45 | -.21 |
|  |  | 4 | -.489* | .080 | .000 | -.65 | -.33 |
|  |  | 5 | -.558* | .084 | .000 | -.72 | -.39 |
|  | 3 | 1 | .813* | .087 | .000 | .64 | .98 |
|  |  | 2 | .333* | .062 | .000 | .21 | .45 |
|  |  | 4 | -.156 | .082 | .056 | -.32 | .00 |
|  |  | 5 | -.225* | .085 | .008 | -.39 | -.06 |
|  | 4 | 1 | .970* | .100 | .000 | .77 | 1.17 |
|  |  | 2 | .489* | .080 | .000 | .33 | .65 |
|  |  | 3 | .156 | .082 | .056 | .00 | .32 |
|  |  | 5 | -.069 | .099 | .488 | -.26 | .13 |
|  | 5 | 1 | 1.039* | .103 | .000 | .84 | 1.24 |
|  |  | 2 | .558* | .084 | .000 | .39 | .72 |
|  |  | 3 | .225* | .085 | .008 | .06 | .39 |
|  |  | 4 | .069 | .099 | .488 | -.13 | .26 |
| BSMAS  Score | 1 | 2 | -1.106* | .282 | .000 | -1.66 | -.55 |
|  |  | 3 | -2.561* | .288 | .000 | -3.13 | -2.00 |
|  |  | 4 | -2.843* | .335 | .000 | -3.50 | -2.19 |
|  |  | 5 | -3.828* | .344 | .000 | -4.50 | -3.15 |
|  | 2 | 1 | 1.106* | .282 | .000 | .55 | 1.66 |
|  |  | 3 | -1.455* | .206 | .000 | -1.86 | -1.05 |
|  |  | 4 | -1.738* | .266 | .000 | -2.26 | -1.22 |
|  |  | 5 | -2.722* | .279 | .000 | -3.27 | -2.18 |
|  | 3 | 1 | 2.561* | .288 | .000 | 2.00 | 3.13 |
|  |  | 2 | 1.455* | .206 | .000 | 1.05 | 1.86 |
|  |  | 4 | -.282 | .273 | .301 | -.82 | .25 |
|  |  | 5 | -1.267* | .285 | .000 | -1.83 | -.71 |
|  | 4 | 1 | 2.843* | .335 | .000 | 2.19 | 3.50 |
|  |  | 2 | 1.738* | .266 | .000 | 1.22 | 2.26 |
|  |  | 3 | .282 | .273 | .301 | -.25 | .82 |
|  |  | 5 | -.985* | .331 | .003 | -1.63 | -.33 |
|  | 5 | 1 | 3.828* | .344 | .000 | 3.15 | 4.50 |
|  |  | 2 | 2.722* | .279 | .000 | 2.18 | 3.27 |
|  |  | 3 | 1.267* | .285 | .000 | .71 | 1.83 |
|  |  | 4 | .985* | .331 | .003 | .33 | 1.63 |
| Social  Media  Addiction | 1 | 2 | .010 | .013 | .435 | -.02 | .04 |
|  |  | 3 | -.012 | .014 | .382 | -.04 | .01 |
|  |  | 4 | -.021 | .016 | .191 | -.05 | .01 |
|  |  | 5 | -.075* | .016 | .000 | -.11 | -.04 |
|  | 2 | 1 | -.010 | .013 | .435 | -.04 | .02 |
|  |  | 3 | -.022* | .010 | .021 | -.04 | .00 |
|  |  | 4 | -.031* | .013 | .014 | -.06 | -.01 |
|  |  | 5 | -.085* | .013 | .000 | -.11 | -.06 |
|  | 3 | 1 | .012 | .014 | .382 | -.01 | .04 |
|  |  | 2 | .022* | .010 | .021 | .00 | .04 |
|  |  | 4 | -.009 | .013 | .498 | -.03 | .02 |
|  |  | 5 | -.063* | .013 | .000 | -.09 | -.04 |
|  | 4 | 1 | .021 | .016 | .191 | -.01 | .05 |
|  |  | 2 | .031* | .013 | .014 | .01 | .06 |
|  |  | 3 | .009 | .013 | .498 | -.02 | .03 |
|  |  | 5 | -.054* | .016 | .001 | -.08 | -.02 |
|  | 5 | 1 | .075* | .016 | .000 | .04 | .11 |
|  |  | 2 | .085* | .013 | .000 | .06 | .11 |
|  |  | 3 | .063* | .013 | .000 | .04 | .09 |
|  |  | 4 | .054* | .016 | .001 | .02 | .08 |

*. The mean difference is significant at the 0.05 level.

## Supplement Table S2 Gender Distribution Comparisons Across Screen Time Groups

| Comparation Groups | Male (Group 1) n (%) | Female (Group 1) n (%) | Male (Group 2) n (%) | Female (Group 2) n (%) | χ² | p |
| --- | --- | --- | --- | --- | --- | --- |
| ≤1h vs 1-2h | 225 (55.69%) | 179 (44.31%) | 741 (58.99%) | 515 (41.01%) | 1.234 | 0.267 |
| ≤1h vs 2-3h | 225 (55.69%) | 179 (44.31%) | 387 (36.20%) | 682 (63.80%) | 15.678 | <0.001 |
| ≤1h vs 3-4h | 225 (55.69%) | 179 (44.31%) | 174 (36.79%) | 299 (63.21%) | 14.567 | <0.001 |
| ≤1h vs >4h | 225 (55.69%) | 179 (44.31%) | 167 (39.86%) | 252 (60.14%) | 12.345 | <0.001 |
| 1-2h vs 2-3h | 741 (58.99%) | 515 (41.01%) | 387 (36.20%) | 682 (63.80%) | 18.765 | <0.001 |
| 1-2h vs 3-4h | 741 (58.99%) | 515 (41.01%) | 174 (36.79%) | 299 (63.21%) | 16.543 | <0.001 |
| 1-2h vs >4h | 741 (58.99%) | 515 (41.01%) | 167 (39.86%) | 252 (60.14%) | 14.567 | <0.001 |
| 2-3h vs 3-4h | 387 (36.20%) | 682 (63.80%) | 174 (36.79%) | 299 (63.21%) | 0.012 | 0.913 |
| 2-3h vs >4h | 387 (36.20%) | 682 (63.80%) | 167 (39.86%) | 252 (60.14%) | 0.345 | 0.557 |
| 3-4h vs >4h | 174 (36.79%) | 299 (63.21%) | 167 (39.86%) | 252 (60.14%) | 0.123 | 0.725 |

Notes:

Group sample sizes: Embedded in group labels (e.g., ≤1h group: N=404; 1-2h group: N=1256).

p-values: Reported as p < 0.001 instead of 0.000 for statistical precision.

Analysis: All comparisons used Pearson’s χ² test with 2×2 contingency tables (df=1).

Group: Group 1 means the first group presented in the “Comparation Groups”, and Group 2 means the second.

## Supplement Table S3 Health Problem Comparisons Across Screen Time Groups

| Comparison Groups | Health Problem (Yes) - Group 1 n (%) | Health Problem (No) - Group 1 n (%) | Health Problem (Yes) - Group 2 n (%) | Health Problem (No) - Group 2 n (%) | χ² | p |
| --- | --- | --- | --- | --- | --- | --- |
| ≤1h vs 1-2h | 153 (37.87%) | 251 (62.13%) | 334 (26.59%) | 922 (73.41%) | 8.765 | 0.003* |
| ≤1h vs 2-3h | 153 (37.87%) | 251 (62.13%) | 308 (28.81%) | 761 (71.19%) | 6.543 | 0.011* |
| ≤1h vs 3-4h | 153 (37.87%) | 251 (62.13%) | 121 (25.58%) | 352 (74.42%) | 12.345 | <0.001* |
| ≤1h vs >4h | 153 (37.87%) | 251 (62.13%) | 149 (35.56%) | 270 (64.44%) | 0.123 | 0.725 |
| 1-2h vs 2-3h | 334 (26.59%) | 922 (73.41%) | 308 (28.81%) | 761 (71.19%) | 0.234 | 0.628 |
| 1-2h vs 3-4h | 334 (26.59%) | 922 (73.41%) | 121 (25.58%) | 352 (74.42%) | 0.012 | 0.913 |
| 1-2h vs >4h | 334 (26.59%) | 922 (73.41%) | 149 (35.56%) | 270 (64.44%) | 4.567 | 0.033* |
| 2-3h vs 3-4h | 308 (28.81%) | 761 (71.19%) | 121 (25.58%) | 352 (74.42%) | 0.789 | 0.374 |
| 2-3h vs >4h | 308 (28.81%) | 761 (71.19%) | 149 (35.56%) | 270 (64.44%) | 2.345 | 0.126 |
| 3-4h vs >4h | 121 (25.58%) | 352 (74.42%) | 149 (35.56%) | 270 (64.44%) | 4.567 | 0.033* |

Notes:

Group sample sizes: Embedded in group labels (e.g., ≤1h group: N=404; 1-2h group: N=1256).

p-values: Reported as p < 0.001 instead of 0.000 for statistical precision.

Analysis: All comparisons used Pearson’s χ² test with 2×2 contingency tables (df=1).

Group: Group 1 means the first group presented in the “Comparation Groups”, and Group 2 means the second.

## Supplement Table S4 Psychiatry History Comparisons Across Screen Time Groups

| Comparison Groups | Psychiatric History (Yes) - Group 1 n (%) | Psychiatric History (No) - Group 1 n (%) | Psychiatric History (Yes) - Group 2 n (%) | Psychiatric History (No) - Group 2 n (%) | χ² | p |
| --- | --- | --- | --- | --- | --- | --- |
| ≤1h vs 1-2h | 104 (25.74%) | 300 (74.26%) | 213 (16.96%) | 1043 (83.04%) | 12.345 | <0.001* |
| ≤1h vs 2-3h | 104 (25.74%) | 300 (74.26%) | 193 (18.06%) | 876 (81.94%) | 6.543 | 0.011* |
| ≤1h vs 3-4h | 104 (25.74%) | 300 (74.26%) | 88 (18.60%) | 385 (81.40%) | 4.567 | 0.033* |
| ≤1h vs >4h | 104 (25.74%) | 300 (74.26%) | 97 (23.15%) | 322 (76.85%) | 0.456 | 0.500 |
| 1-2h vs 2-3h | 213 (16.96%) | 1043 (83.04%) | 193 (18.06%) | 876 (81.94%) | 0.234 | 0.628 |
| 1-2h vs 3-4h | 213 (16.96%) | 1043 (83.04%) | 88 (18.60%) | 385 (81.40%) | 0.234 | 0.628 |
| 1-2h vs >4h | 213 (16.96%) | 1043 (83.04%) | 97 (23.15%) | 322 (76.85%) | 4.567 | 0.033* |
| 2-3h vs 3-4h | 193 (18.06%) | 876 (81.94%) | 88 (18.60%) | 385 (81.40%) | 0.012 | 0.913 |
| 2-3h vs >4h | 193 (18.06%) | 876 (81.94%) | 97 (23.15%) | 322 (76.85%) | 2.345 | 0.126 |
| 3-4h vs >4h | 88 (18.60%) | 385 (81.40%) | 97 (23.15%) | 322 (76.85%) | 1.234 | 0.267 |

Notes:

Group sample sizes: Embedded in group labels (e.g., ≤1h group: N=404; 1-2h group: N=1256).

p-values: Reported as p < 0.001 instead of 0.000 for statistical precision.

Analysis: All comparisons used Pearson’s χ² test with 2×2 contingency tables (df=1).

Group: Group 1 means the first group presented in the “Comparation Groups”, and Group 2 means the second.

## Supplement Table S5 Consultant History Comparisons Across Screen Time Groups

| Comparison Groups | Consultant History (Yes) - Group 1 n (%) | Consultant History (No) - Group 1 n (%) | Consultant History (Yes) - Group 2 n (%) | Consultant History (No) - Group 2 n (%) | χ² | p |
| --- | --- | --- | --- | --- | --- | --- |
| ≤1h vs 1-2h | 48 (11.88%) | 356 (88.12%) | 146 (11.62%) | 1110 (88.38%) | 0.012 | 0.913 |
| ≤1h vs 2-3h | 48 (11.88%) | 356 (88.12%) | 150 (14.03%) | 919 (85.97%) | 1.234 | 0.267 |
| ≤1h vs 3-4h | 48 (11.88%) | 356 (88.12%) | 69 (14.59%) | 404 (85.41%) | 1.234 | 0.267 |
| ≤1h vs >4h | 48 (11.88%) | 356 (88.12%) | 59 (14.08%) | 360 (85.92%) | 0.789 | 0.374 |
| 1-2h vs 2-3h | 146 (11.62%) | 1110 (88.38%) | 150 (14.03%) | 919 (85.97%) | 2.345 | 0.126 |
| 1-2h vs 3-4h | 146 (11.62%) | 1110 (88.38%) | 69 (14.59%) | 404 (85.41%) | 1.234 | 0.267 |
| 1-2h vs >4h | 146 (11.62%) | 1110 (88.38%) | 59 (14.08%) | 360 (85.92%) | 1.234 | 0.267 |
| 2-3h vs 3-4h | 150 (14.03%) | 919 (85.97%) | 69 (14.59%) | 404 (85.41%) | 0.056 | 0.813 |
| 2-3h vs >4h | 150 (14.03%) | 919 (85.97%) | 59 (14.08%) | 360 (85.92%) | 0.001 | 0.978 |
| 3-4h vs >4h | 69 (14.59%) | 404 (85.41%) | 59 (14.08%) | 360 (85.92%) | 0.056 | 0.813 |

Notes:

Group sample sizes: Embedded in group labels (e.g., ≤1h group: N=404; 1-2h group: N=1256).

p-values: Reported as p < 0.001 instead of 0.000 for statistical precision.

Analysis: All comparisons used Pearson’s χ² test with 2×2 contingency tables (df=1).

Group: Group 1 means the first group presented in the “Comparation Groups”, and Group 2 means the second.

## Supplement Table S6 Single Child Condition Comparisons Across Screen Time Groups

| Comparison Groups | Single Child (Yes) - Group 1 n (%) | Single Child (No) - Group 1 n (%) | Single Child (Yes) - Group 2 n (%) | Single Child (No) - Group 2 n (%) | χ² | p |
| --- | --- | --- | --- | --- | --- | --- |
| ≤1h vs 1-2h | 239 (59.16%) | 165 (40.84%) | 747 (59.48%) | 509 (40.52%) | 0.012 | 0.913 |
| ≤1h vs 2-3h | 239 (59.16%) | 165 (40.84%) | 613 (57.34%) | 456 (42.66%) | 0.234 | 0.628 |
| ≤1h vs 3-4h | 239 (59.16%) | 165 (40.84%) | 290 (61.31%) | 183 (38.69%) | 0.345 | 0.557 |
| ≤1h vs >4h | 239 (59.16%) | 165 (40.84%) | 255 (60.86%) | 164 (39.14%) | 0.123 | 0.725 |
| 1-2h vs 2-3h | 747 (59.48%) | 509 (40.52%) | 613 (57.34%) | 456 (42.66%) | 0.567 | 0.451 |
| 1-2h vs 3-4h | 747 (59.48%) | 509 (40.52%) | 290 (61.31%) | 183 (38.69%) | 0.345 | 0.557 |
| 1-2h vs >4h | 747 (59.48%) | 509 (40.52%) | 255 (60.86%) | 164 (39.14%) | 0.123 | 0.725 |
| 2-3h vs 3-4h | 613 (57.34%) | 456 (42.66%) | 290 (61.31%) | 183 (38.69%) | 0.789 | 0.374 |
| 2-3h vs >4h | 613 (57.34%) | 456 (42.66%) | 255 (60.86%) | 164 (39.14%) | 0.567 | 0.451 |
| 3-4h vs >4h | 290 (61.31%) | 183 (38.69%) | 255 (60.86%) | 164 (39.14%) | 0.012 | 0.913 |

Notes:

Group sample sizes: Embedded in group labels (e.g., ≤1h group: N=404; 1-2h group: N=1256).

p-values: Reported as p < 0.001 instead of 0.000 for statistical precision.

Analysis: All comparisons used Pearson’s χ² test with 2×2 contingency tables (df=1).

Group: Group 1 means the first group presented in the “Comparation Groups”, and Group 2 means the second.

## Supplement Table S7 Financial Burden Comparisons Across Screen Time Groups

| Comparison Groups | Financial Burden (Yes) - Group 1 n (%) | Financial Burden (No) - Group 1 n (%) | Financial Burden (Yes) - Group 2 n (%) | Financial Burden (No) - Group 2 n (%) | χ² | p |
| --- | --- | --- | --- | --- | --- | --- |
| ≤1h vs 1-2h | 179 (44.31%) | 225 (55.69%) | 412 (32.80%) | 844 (67.20%) | 15.678 | <0.001* |
| ≤1h vs 2-3h | 179 (44.31%) | 225 (55.69%) | 309 (28.90%) | 760 (71.10%) | 23.456 | <0.001* |
| ≤1h vs 3-4h | 179 (44.31%) | 225 (55.69%) | 143 (30.23%) | 330 (69.77%) | 18.765 | <0.001* |
| ≤1h vs >4h | 179 (44.31%) | 225 (55.69%) | 133 (31.74%) | 286 (68.26%) | 14.567 | <0.001* |
| 1-2h vs 2-3h | 412 (32.80%) | 844 (67.20%) | 309 (28.90%) | 760 (71.10%) | 2.345 | 0.126 |
| 1-2h vs 3-4h | 412 (32.80%) | 844 (67.20%) | 143 (30.23%) | 330 (69.77%) | 0.567 | 0.451 |
| 1-2h vs >4h | 412 (32.80%) | 844 (67.20%) | 133 (31.74%) | 286 (68.26%) | 0.123 | 0.725 |
| 2-3h vs 3-4h | 309 (28.90%) | 760 (71.10%) | 143 (30.23%) | 330 (69.77%) | 0.123 | 0.725 |
| 2-3h vs >4h | 309 (28.90%) | 760 (71.10%) | 133 (31.74%) | 286 (68.26%) | 0.345 | 0.557 |
| 3-4h vs >4h | 143 (30.23%) | 330 (69.77%) | 133 (31.74%) | 286 (68.26%) | 0.123 | 0.725 |

Notes:

Group sample sizes: Embedded in group labels (e.g., ≤1h group: N=404; 1-2h group: N=1256).

p-values: Reported as p < 0.001 instead of 0.000 for statistical precision.

Analysis: All comparisons used Pearson’s χ² test with 2×2 contingency tables (df=1).

Group: Group 1 means the first group presented in the “Comparation Groups”, and Group 2 means the second.

## Supplement Table S8 Social Media Addiction Comparisons Across Screen Time Groups

| Comparison Groups | Media Addiction (Yes) - Group 1 n (%) | Media Addiction (No) - Group 1 n (%) | Media Addiction (Yes) - Group 2 n (%) | Media Addiction (No) - Group 2 n (%) | χ² | p |
| --- | --- | --- | --- | --- | --- | --- |
| ≤1h vs 1-2h | 19 (4.70%) | 385 (95.30%) | 46 (3.66%) | 1210 (96.34%) | 1.234 | 0.267 |
| ≤1h vs 2-3h | 19 (4.70%) | 385 (95.30%) | 63 (5.89%) | 1006 (94.11%) | 0.987 | 0.321 |
| ≤1h vs 3-4h | 19 (4.70%) | 385 (95.30%) | 32 (6.77%) | 441 (93.23%) | 1.234 | 0.267 |
| ≤1h vs >4h | 19 (4.70%) | 385 (95.30%) | 51 (12.20%) | 369 (87.80%) | 12.345 | <0.001* |
| 1-2h vs 2-3h | 46 (3.66%) | 1210 (96.34%) | 63 (5.89%) | 1006 (94.11%) | 4.567 | 0.033* |
| 1-2h vs 3-4h | 46 (3.66%) | 1210 (96.34%) | 32 (6.77%) | 441 (93.23%) | 3.456 | 0.063 |
| 1-2h vs >4h | 46 (3.66%) | 1210 (96.34%) | 51 (12.20%) | 369 (87.80%) | 16.543 | <0.001* |
| 2-3h vs 3-4h | 63 (5.89%) | 1006 (94.11%) | 32 (6.77%) | 441 (93.23%) | 0.234 | 0.628 |
| 2-3h vs >4h | 63 (5.89%) | 1006 (94.11%) | 51 (12.20%) | 369 (87.80%) | 9.876 | 0.002* |
| 3-4h vs >4h | 32 (6.77%) | 441 (93.23%) | 51 (12.20%) | 369 (87.80%) | 5.678 | 0.017* |

Notes:

Group sample sizes: Embedded in group labels (e.g., ≤1h group: N=404; 1-2h group: N=1256).

p-values: Reported as p < 0.001 instead of 0.000 for statistical precision.

Analysis: All comparisons used Pearson’s χ² test with 2×2 contingency tables (df=1).

Group: Group 1 means the first group presented in the “Comparation Groups”, and Group 2 means the second.

**Supplement Table S9 ANOVA and Post-hoc Results for Platform Numbers and BSMAS by Training Year**

| **Groups** | **Mean Difference** | **Std. Error** | **Sig.** | **95% CI** |
| --- | --- | --- | --- | --- |
| **Platform Numbers** |  |  |  |  |
| 1 vs 2 | -0.015 | 0.058 | 0.793 | [-0.13, 0.10] |
| 1 vs 3 | 0.100 | 0.065 | 0.126 | [-0.03, 0.23] |
| 2 vs 3 | 0.115 | 0.069 | 0.098 | [-0.02, 0.25] |
| **BSMAS Score** |  |  |  |  |
| 1 vs 2 | -0.788* | 0.194 | 0.000 | [-1.17, -0.41] |
| 1 vs 3 | -0.502* | 0.217 | 0.021 | [-0.93, -0.08] |
| 2 vs 3 | 0.286 | 0.232 | 0.218 | [-0.17, 0.74] |

The tables present LSD comparison results for platform numbers and BSMAS scores across different training years. "Groups" indicate the years being compared (e.g., 1 vs 2). Key metrics include Mean Difference, Std. Error, Sig. (p-value), and 95% CI. BSMAS refers to the Bergen Social Media Addiction Scale. Significant results are marked with an asterisk (*), indicating significance at the 0.05 level.

## Supplement Table S10 Univariate Regression Models for Social Media Addiction

| **Variable** | **estimate** | **SD** | **statistic** | **p** | **conf.low** | **conf.high** |
| --- | --- | --- | --- | --- | --- | --- |
| (Intercept) | 0.004 | 0.859 | -6.353 | 0.000 | 0.001 | 0.023 |
| Age | 1.006 | 0.031 | 0.200 | 0.842 | 0.945 | 1.068 |
| Night Shifts per Month | 1.018 | 0.012 | 1.457 | 0.145 | 0.993 | 1.041 |
| Occupational Burnout | 1.409 | 0.071 | 4.848 | 0.000 | 1.226 | 1.617 |
| Openness | 0.984 | 0.039 | -0.411 | 0.681 | 0.911 | 1.062 |
| Conscientiousness | 0.922 | 0.036 | -2.277 | 0.023 | 0.860 | 0.989 |
| Extraversion | 1.008 | 0.030 | 0.274 | 0.784 | 0.950 | 1.069 |
| Agreeableness | 0.992 | 0.038 | -0.211 | 0.833 | 0.921 | 1.069 |
| Stability | 0.941 | 0.038 | -1.611 | 0.107 | 0.874 | 1.013 |
| Frequently Used Platform Numbers | 1.086 | 0.045 | 1.845 | 0.065 | 0.993 | 1.184 |
| Social Media Time per Day | 1.388 | 0.060 | 5.433 | 0.000 | 1.233 | 1.562 |
| Sex | 0.884 | 0.155 | -0.794 | 0.427 | 0.653 | 1.200 |
| Physical Health Problems | 1.564 | 0.164 | 2.728 | 0.006 | 1.133 | 2.157 |
| Psychiatric History | 2.000 | 0.179 | 3.871 | 0.000 | 1.406 | 2.838 |
| Psychological Consultant History | 1.393 | 0.187 | 1.779 | 0.075 | 0.962 | 2.000 |
| Single Child | 0.862 | 0.155 | -0.957 | 0.339 | 0.636 | 1.171 |
| Financial Burden | 1.122 | 0.162 | 0.708 | 0.479 | 0.814 | 1.538 |

This univariate regression analysis evaluates social media addiction assessed using the BAMAS scale. The table lists each predictor variable along with its estimated coefficient, standard error, test statistic, p-value, and the 95% confidence interval. Variables with a p-value less than 0.05 are considered statistically significant predictors of social media addiction.

## Supplement Table S11 Logistic Regression Models for Social Media Addiction

| **Variable** | **estimate** | **SD** | **statistic** | **p** | **conf.low** | **conf.high** |
| --- | --- | --- | --- | --- | --- | --- |
| (Intercept) | 0.004 | 0.859 | -6.353 | 0.000 | 0.001 | 0.023 |
| Age | 1.006 | 0.031 | 0.200 | 0.842 | 0.945 | 1.068 |
| Night Shifts per Month | 1.018 | 0.012 | 1.457 | 0.145 | 0.993 | 1.041 |
| Occupational Burnout | 1.409 | 0.071 | 4.848 | 0.000 | 1.226 | 1.617 |
| Openness | 0.984 | 0.039 | -0.411 | 0.681 | 0.911 | 1.062 |
| Conscientiousness | 0.922 | 0.036 | -2.277 | 0.023 | 0.860 | 0.989 |
| Extraversion | 1.008 | 0.030 | 0.274 | 0.784 | 0.950 | 1.069 |
| Agreeableness | 0.992 | 0.038 | -0.211 | 0.833 | 0.921 | 1.069 |
| Stability | 0.941 | 0.038 | -1.611 | 0.107 | 0.874 | 1.013 |
| Frequently Used Platform Numbers | 1.086 | 0.045 | 1.845 | 0.065 | 0.993 | 1.184 |
| Social Media Time per Day | 1.388 | 0.060 | 5.433 | 0.000 | 1.233 | 1.562 |
| Sex | 0.884 | 0.155 | -0.794 | 0.427 | 0.653 | 1.200 |
| Physical Health Problems | 1.564 | 0.164 | 2.728 | 0.006 | 1.133 | 2.157 |
| Psychiatric History | 2.000 | 0.179 | 3.871 | 0.000 | 1.406 | 2.838 |
| Psychological Consultant History | 1.393 | 0.187 | 1.779 | 0.075 | 0.962 | 2.000 |
| Single Child | 0.862 | 0.155 | -0.957 | 0.339 | 0.636 | 1.171 |
| Financial Burden | 1.122 | 0.162 | 0.708 | 0.479 | 0.814 | 1.538 |

This logistic regression analysis evaluates social media addiction assessed using the BAMAS scale, with a cutoff score of 24 used to define addiction. The table lists each predictor variable along with its estimated coefficient, standard error, test statistic, p-value, and the 95% confidence interval. Variables with a p-value less than 0.05 are considered statistically significant predictors of social media addiction.

**Supplement Table S12 Mediation Analysis of Stable Predictors on Social Media Addiction via Occupational Burnout and Social Media Time per Day**

| **Mediation Models** | **X→M** | | **M→Y** | | **X→Y** | | **Total Effect** | **Direct Effect** | **Mediation effect** | | | | | **Mediation Proportion** |
| --- | --- | --- | --- | --- | --- | --- | --- | --- | --- | --- | --- | --- | --- | --- |
|  | ***β*** | ***p*-value** | ***β*** | ***p*-value** | ***β*** | ***p*-value** | ***p*-value** | ***p*-value** | ***β*** | ***p*-value** | **BootSE** | **BootLLCI** | **BootULCI** |  |
| **Mediator: Occupational Burnout** | | | | | | | | | | | | | | |
| Psychiatric History | 0.809 | <0.001 | 0.898 | <0.001 | 1.855 | <0.001 | <0.001 | <0.001 | 0.726 | <0.001 | 0.090 | 0.558 | 0.909 | 0.281 |
| Health Problems | 0.681 | <0.001 | 0.919 | <0.001 | 1.485 | <0.001 | <0.001 | <0.001 | 0.626 | <0.001 | 0.074 | 0.484 | 0.779 | 0.296 |
| Conscientiousness | -0.119 | <0.001 | 0.872 | <0.001 | -0.395 | <0.001 | <0.001 | <0.001 | -0.104 | <0.001 | 0.013 | -0.131 | -0.080 | 0.209 |
| **Mediator: Social Media Time per Day** | | | | | | | | | | | | | | |
| Psychiatric History | 0.01 | 0.847 | 0.950 | <0.001 | 2.572 | <0.001 | <0.001 | <0.001 | 0.009 | 0.847 | 0.049 | -0.085 | 0.105 | 0.004 |
| Health Problems | 0.001 | 0.989 | 0.953 | <0.001 | 2.110 | <0.001 | <0.001 | <0.001 | 0.001 | 0.989 | 0.042 | -0.084 | 0.085 | <0.001 |
| Conscientiousness | -0.023 | 0.008 | 0.908 | <0.001 | -0.478 | <0.001 | <0.001 | <0.001 | -0.021 | 0.009 | 0.008 | -0.037 | -0.006 | 0.041 |

This table shows the mediation analysis results for the effects of Psychiatric History, Physical Health Problems, and Conscientiousness on social media addiction (measured by BSMAS scores) through two mediators: Occupational Burnout and Social Media Time per Day. For each model, standardized coefficients (β) and p-values are provided for the paths from the predictor to the mediator (X→M), from the mediator to the outcome (M→Y), and the direct effect (X→Y). In addition, the total effect, indirect (mediation) effect with its bootstrap (5000 times) standard error and 95% confidence interval, and the mediation proportion are reported. All statistics are rounded to three decimal places.

## Supplement Table S13 Moderation Analysis of Stable Predictors on BSMAS Scores by Social Media Time and Occupational Burnout

| **Moderation Models** | **X→Y** | | **M→Y** | | **X*M** | | **R^2^** |
| --- | --- | --- | --- | --- | --- | --- | --- |
|  | ***β*** | ***p*-value** | ***β*** | ***p*-value** | ***β*** | ***p*-value** |  |
| **Moderator: Occupational Burnout** | | | | | | | |
| Psychiatric History | 4.076 | <0.001 | 1.909 | <0.001 | -0.795 | <0.001 | 0.075 |
| Health Problems | 2.804 | <0.001 | 1.635 | <0.001 | -0.513 | 0.002 | 0.07 |
| Conscientiousness | -0.462 | <0.001 | 0.858 | <0.001 | 0.028 | 0.341 | 0.083 |
| **Moderator: Social Media Time per Day** | | | | | | | |
| Psychiatric History | 2.772 | <0.001 | 0.919 | <0.001 | -0.071 | 0.631 | 0.087 |
| Health Problems | 2.228 | <0.001 | 0.893 | <0.001 | -0.046 | 0.726 | 0.082 |
| Conscientiousness | -0.504 | <0.001 | 0.788 | <0.001 | 0.009 | 0.712 | 0.096 |

This table presents the results of moderation analyses examining the interaction effects between each stable predictor—Psychiatric History, Physical Health Problems, and Conscientiousness—and the dynamic moderators (Social Media Time and Occupational Burnout) on BSMAS scores. For each model, the table reports the standardized regression coefficient (β) and corresponding p-value for the effect of the stable predictor (X), the moderator (M), and their interaction (X×M), as well as the model’s overall R². Significant interaction effects indicate that the moderator meaningfully alters the relationship between the stable predictor and BSMAS scores.

## Supplement Table S14 Logistic Regression Models for Social Media Addiction in First Year Trainees

| **Variable** | **estimate** | **SD** | **statistic** | **p** | **conf.low** | **conf.high** |
| --- | --- | --- | --- | --- | --- | --- |
| (Intercept) | 0.006 | 1.703 | -2.961 | 0.003 | 0.000 | 0.206 |
| Age | 0.979 | 0.065 | -0.324 | 0.746 | 0.856 | 1.102 |
| Night Shifts per Month | 0.994 | 0.023 | -0.285 | 0.776 | 0.947 | 1.036 |
| Occupational Burnout | 1.495 | 0.132 | 3.043 | 0.002 | 1.151 | 1.934 |
| Openness | 0.879 | 0.076 | -1.699 | 0.089 | 0.758 | 1.019 |
| Conscientiousness | 1.026 | 0.068 | 0.377 | 0.706 | 0.898 | 1.174 |
| Extraversion | 1.016 | 0.056 | 0.277 | 0.782 | 0.908 | 1.133 |
| Agreeableness | 1.004 | 0.068 | 0.065 | 0.948 | 0.879 | 1.150 |
| Stability | 0.928 | 0.071 | -1.048 | 0.295 | 0.807 | 1.067 |
| Frequently Used Platform Numbers | 0.948 | 0.092 | -0.582 | 0.561 | 0.784 | 1.122 |
| Social Media Time per Day | 1.519 | 0.109 | 3.844 | 0.000 | 1.228 | 1.882 |
| Sex | 0.852 | 0.272 | -0.588 | 0.557 | 0.500 | 1.459 |
| Physical Health Problems | 1.816 | 0.290 | 2.060 | 0.039 | 1.025 | 3.203 |
| Psychiatric History | 2.701 | 0.319 | 3.111 | 0.002 | 1.436 | 5.039 |
| Psychological Consultant History | 1.308 | 0.347 | 0.773 | 0.439 | 0.648 | 2.539 |
| Single Child | 0.865 | 0.278 | -0.522 | 0.602 | 0.503 | 1.503 |
| Financial Burden | 1.112 | 0.296 | 0.359 | 0.719 | 0.615 | 1.971 |

This logistic regression analysis evaluates social media addiction assessed using the BAMAS scale in first year trainees, with a cutoff score of 24 used to define addiction. The table lists each predictor variable along with its estimated coefficient, standard error, test statistic, p-value, and the 95% confidence interval. Variables with a p-value less than 0.05 are considered statistically significant predictors of social media addiction.

## Supplement Table S15 Logistic Regression Models for Social Media Addiction in Second Year Trainees

| **Variable** | **estimate** | **SD** | **statistic** | **p** | **conf.low** | **conf.high** |
| --- | --- | --- | --- | --- | --- | --- |
| (Intercept) | 0.019 | 1.369 | -2.898 | 0.004 | 0.001 | 0.288 |
| Age | 0.977 | 0.049 | -0.464 | 0.643 | 0.885 | 1.073 |
| Night Shifts per Month | 1.043 | 0.017 | 2.424 | 0.015 | 1.007 | 1.078 |
| Occupational Burnout | 1.344 | 0.109 | 2.722 | 0.006 | 1.084 | 1.661 |
| Openness | 1.082 | 0.060 | 1.314 | 0.189 | 0.962 | 1.217 |
| Conscientiousness | 0.869 | 0.054 | -2.591 | 0.010 | 0.780 | 0.966 |
| Extraversion | 1.046 | 0.046 | 0.965 | 0.335 | 0.955 | 1.146 |
| Agreeableness | 0.925 | 0.060 | -1.314 | 0.189 | 0.822 | 1.039 |
| Stability | 1.013 | 0.056 | 0.236 | 0.813 | 0.909 | 1.130 |
| Frequently Used Platform Numbers | 1.119 | 0.068 | 1.653 | 0.098 | 0.976 | 1.275 |
| Social Media Time per Day | 1.143 | 0.097 | 1.376 | 0.169 | 0.944 | 1.381 |
| Sex | 1.150 | 0.241 | 0.581 | 0.561 | 0.720 | 1.856 |
| Physical Health Problems | 1.241 | 0.257 | 0.843 | 0.399 | 0.748 | 2.049 |
| Psychiatric History | 1.630 | 0.284 | 1.722 | 0.085 | 0.930 | 2.835 |
| Psychological Consultant History | 1.724 | 0.282 | 1.931 | 0.053 | 0.984 | 2.980 |
| Single Child | 0.764 | 0.241 | -1.120 | 0.263 | 0.476 | 1.226 |
| Financial Burden | 1.447 | 0.243 | 1.523 | 0.128 | 0.895 | 2.323 |

This logistic regression analysis evaluates social media addiction assessed using the BAMAS scale in second year trainees, with a cutoff score of 24 used to define addiction. The table lists each predictor variable along with its estimated coefficient, standard error, test statistic, p-value, and the 95% confidence interval. Variables with a p-value less than 0.05 are considered statistically significant predictors of social media addiction.

## Supplement Table S16 Logistic Regression Models for Social Media Addiction in Third Year Trainees

| **Variable** | **estimate** | **SD** | **statistic** | **p** | **conf.low** | **conf.high** |
| --- | --- | --- | --- | --- | --- | --- |
| (Intercept) | 0.000 | 1.864 | -4.440 | 0.000 | 0.000 | 0.010 |
| Age | 1.071 | 0.062 | 1.105 | 0.269 | 0.945 | 1.206 |
| Night Shifts per Month | 0.996 | 0.029 | -0.137 | 0.891 | 0.936 | 1.051 |
| Occupational Burnout | 1.506 | 0.147 | 2.792 | 0.005 | 1.131 | 2.014 |
| Openness | 0.964 | 0.075 | -0.480 | 0.631 | 0.830 | 1.117 |
| Conscientiousness | 0.886 | 0.073 | -1.650 | 0.099 | 0.766 | 1.022 |
| Extraversion | 0.975 | 0.061 | -0.418 | 0.676 | 0.863 | 1.098 |
| Agreeableness | 1.090 | 0.078 | 1.104 | 0.270 | 0.935 | 1.271 |
| Stability | 0.854 | 0.082 | -1.934 | 0.053 | 0.725 | 1.000 |
| Frequently Used Platform Numbers | 1.179 | 0.104 | 1.583 | 0.113 | 0.967 | 1.455 |
| Social Media Time per Day | 1.807 | 0.128 | 4.621 | 0.000 | 1.413 | 2.339 |
| Sex | 0.515 | 0.339 | -1.956 | 0.050 | 0.264 | 1.005 |
| Physical Health Problems | 1.689 | 0.340 | 1.539 | 0.124 | 0.866 | 3.309 |
| Psychiatric History | 2.209 | 0.363 | 2.183 | 0.029 | 1.081 | 4.511 |
| Psychological Consultant History | 0.995 | 0.404 | -0.013 | 0.990 | 0.436 | 2.144 |
| Single Child | 1.107 | 0.331 | 0.307 | 0.759 | 0.583 | 2.146 |
| Financial Burden | 0.559 | 0.358 | -1.625 | 0.104 | 0.272 | 1.111 |

This logistic regression analysis evaluates social media addiction assessed using the BAMAS scale in third year trainees, with a cutoff score of 24 used to define addiction. The table lists each predictor variable along with its estimated coefficient, standard error, test statistic, p-value, and the 95% confidence interval. Variables with a p-value less than 0.05 are considered statistically significant predictors of social media addiction.

## Supplement Table S17. Coefficients and Multicollinearity Diagnostics for Predictors of BSMAS Score

| **Predictor** | **B** | **SE** | **Beta** | **t** | **p-value** | **Tolerance** | **VIF** |
| --- | --- | --- | --- | --- | --- | --- | --- |
| Constant | 11.508 | 0.943 |  | 12.209 | 0.0 |  |  |
| Sex | -0.19 | 0.16 | -0.018 | -1.192 | 0.233 | 0.929 | 1.076 |
| Age | -0.074 | 0.031 | -0.036 | -2.363 | 0.018 | 0.957 | 1.045 |
| Physical Health Problems | 0.806 | 0.184 | 0.072 | 4.369 | 0.0 | 0.81 | 1.235 |
| Psychiatric History | 0.808 | 0.229 | 0.063 | 3.534 | 0.0 | 0.705 | 1.418 |
| Psychological Consultant History | 0.756 | 0.249 | 0.05 | 3.037 | 0.002 | 0.815 | 1.227 |
| Single Child | -0.218 | 0.159 | -0.021 | -1.368 | 0.171 | 0.935 | 1.07 |
| Financial Burden | -0.112 | 0.172 | -0.01 | -0.652 | 0.514 | 0.884 | 1.132 |
| Night Shifts per Month | 0.013 | 0.014 | 0.013 | 0.874 | 0.382 | 0.978 | 1.022 |
| Social media per Day | 0.832 | 0.067 | 0.19 | 12.435 | 0.0 | 0.952 | 1.051 |
| Occupational Burnout | 0.425 | 0.085 | 0.087 | 4.983 | 0.0 | 0.734 | 1.362 |
| Extraversion | 0.045 | 0.032 | 0.023 | 1.419 | 0.156 | 0.816 | 1.226 |
| Stability | -0.255 | 0.041 | -0.122 | -6.252 | 0.0 | 0.589 | 1.697 |
| Openness | -0.136 | 0.042 | -0.059 | -3.257 | 0.001 | 0.673 | 1.486 |
| Agreeableness | -0.236 | 0.042 | -0.097 | -5.593 | 0.0 | 0.742 | 1.347 |
| Conscientiousness | -0.16 | 0.039 | -0.074 | -4.143 | 0.0 | 0.691 | 1.448 |
| F.U. Platform Numbers | 0.347 | 0.052 | 0.104 | 6.704 | 0.0 | 0.931 | 1.074 |

Notes: Dependent variable = BSMAS score. B = Unstandardized regression coefficient; SE = Standard error; Beta = Standardized coefficient; F.U. = Frequently used; VIF = Variance Inflation Factor. All coefficients are from linear regression models including all predictors.

**Supplement Figure S1** **Comparison of Social Media Addiction Proportions Among Residents in Different Training Years**


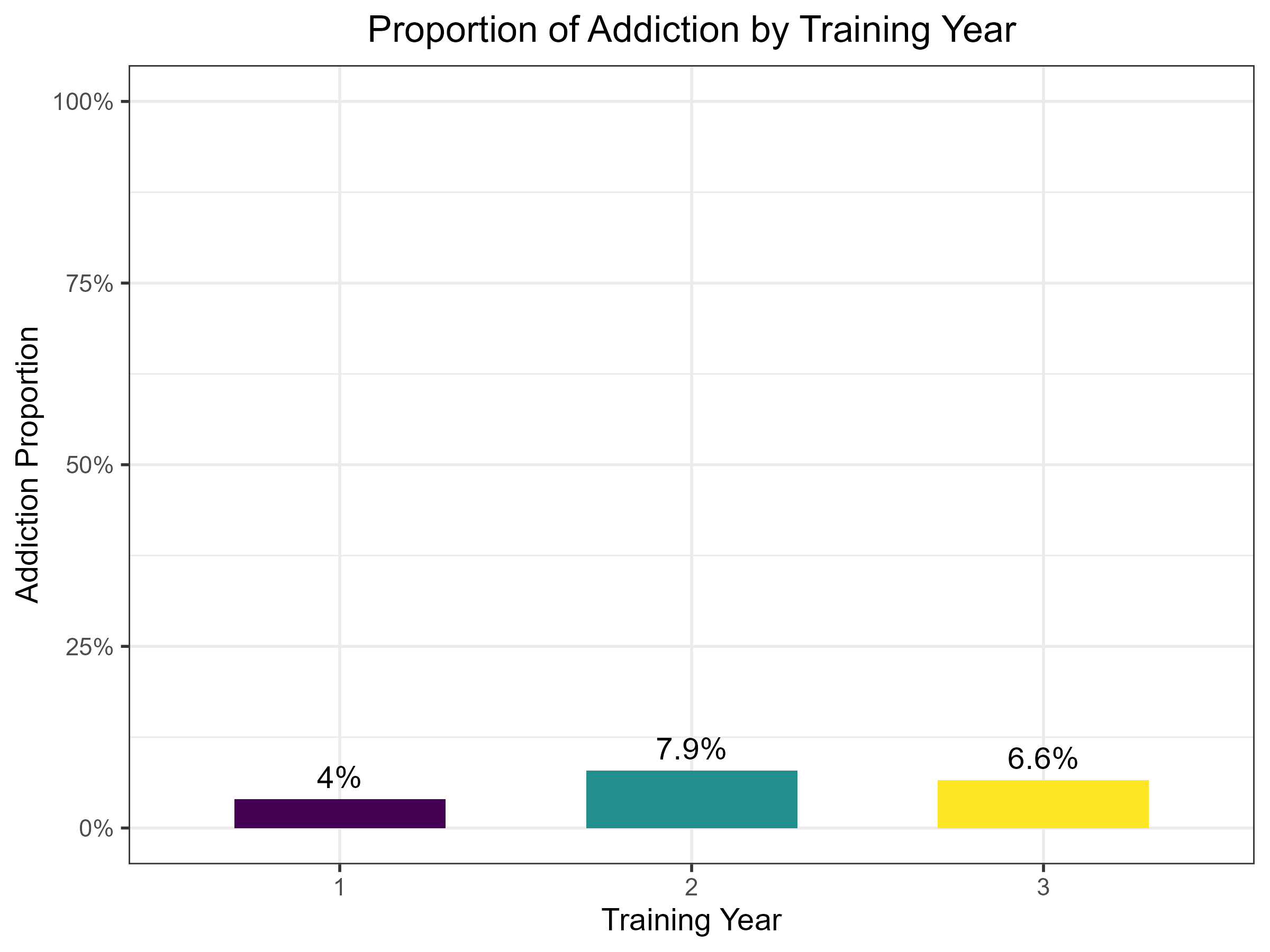


This figure illustrates the results of pairwise comparisons of social media addiction (defined as BSMAS ≥ 24) proportions among residents in different training years (1, 2, and 3). Additional post - hoc analysis indicates that the proportion of social media addiction in Training Year 1 is significantly lower than that in Training Year 2 (Mean Difference = -0.040, Standard Error = 0.009, p = 0.000, 95% CI [-0.06, -0.02]) and Training Year 3 (Mean Difference = -0.026, Standard Error = 0.010, p = 0.010, 95% CI [-0.05, -0.01]).

**Supplement Figure S2** **Number of Frequently Used Platforms Among Residents in Different Training Years**


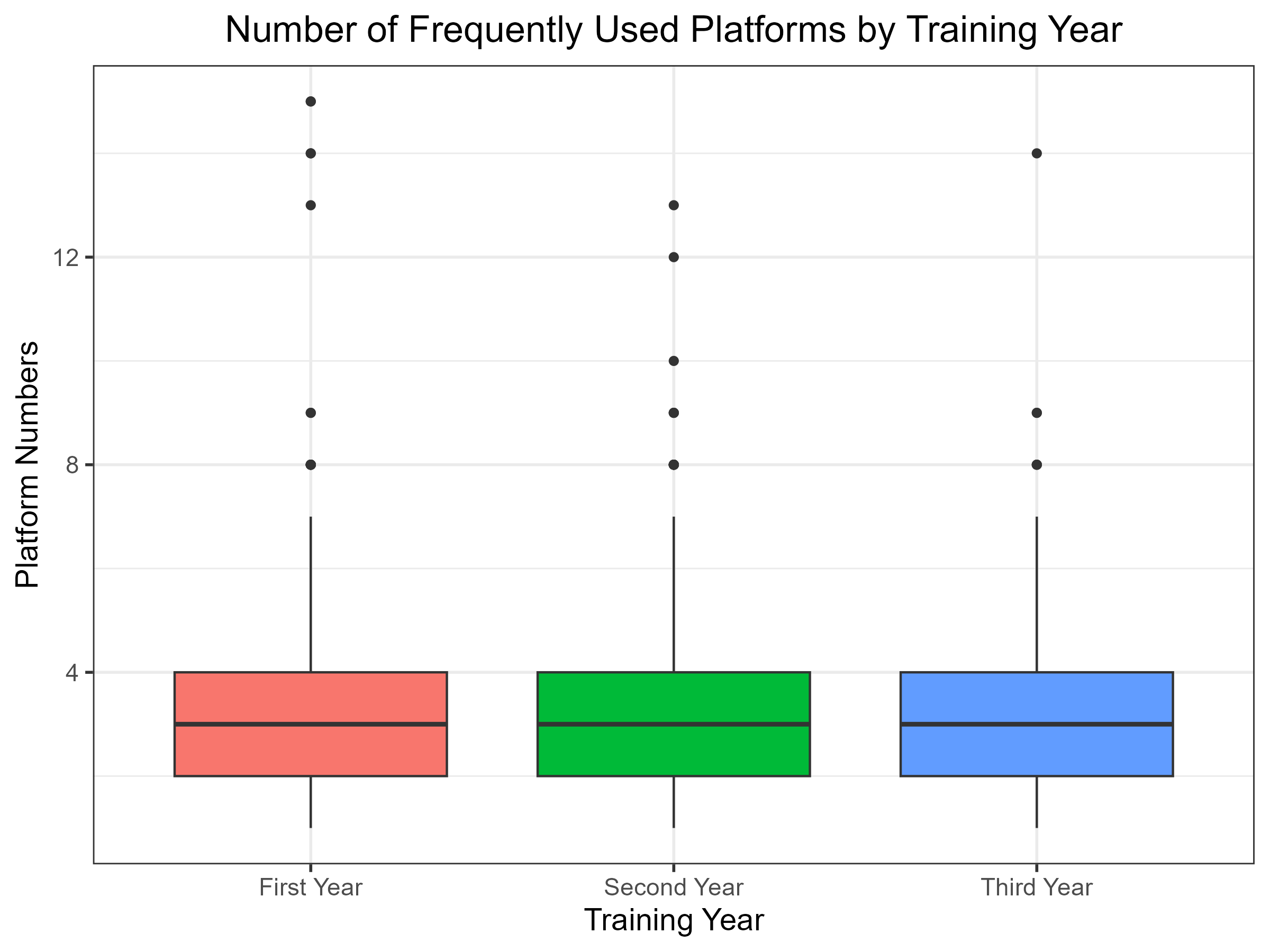


This boxplot illustrates the distribution of the number of frequently used platforms (PlatformNumbers) among resident physicians in their first, second, and third years of training. The central line within each box represents the median, and the upper and lower edges of the box mark the interquartile range. Whiskers and outlier points indicate data variability and potential extremes.

**Supplement Figure S3 Comparison of BSMAS Score Among Residents in Different Training Years**


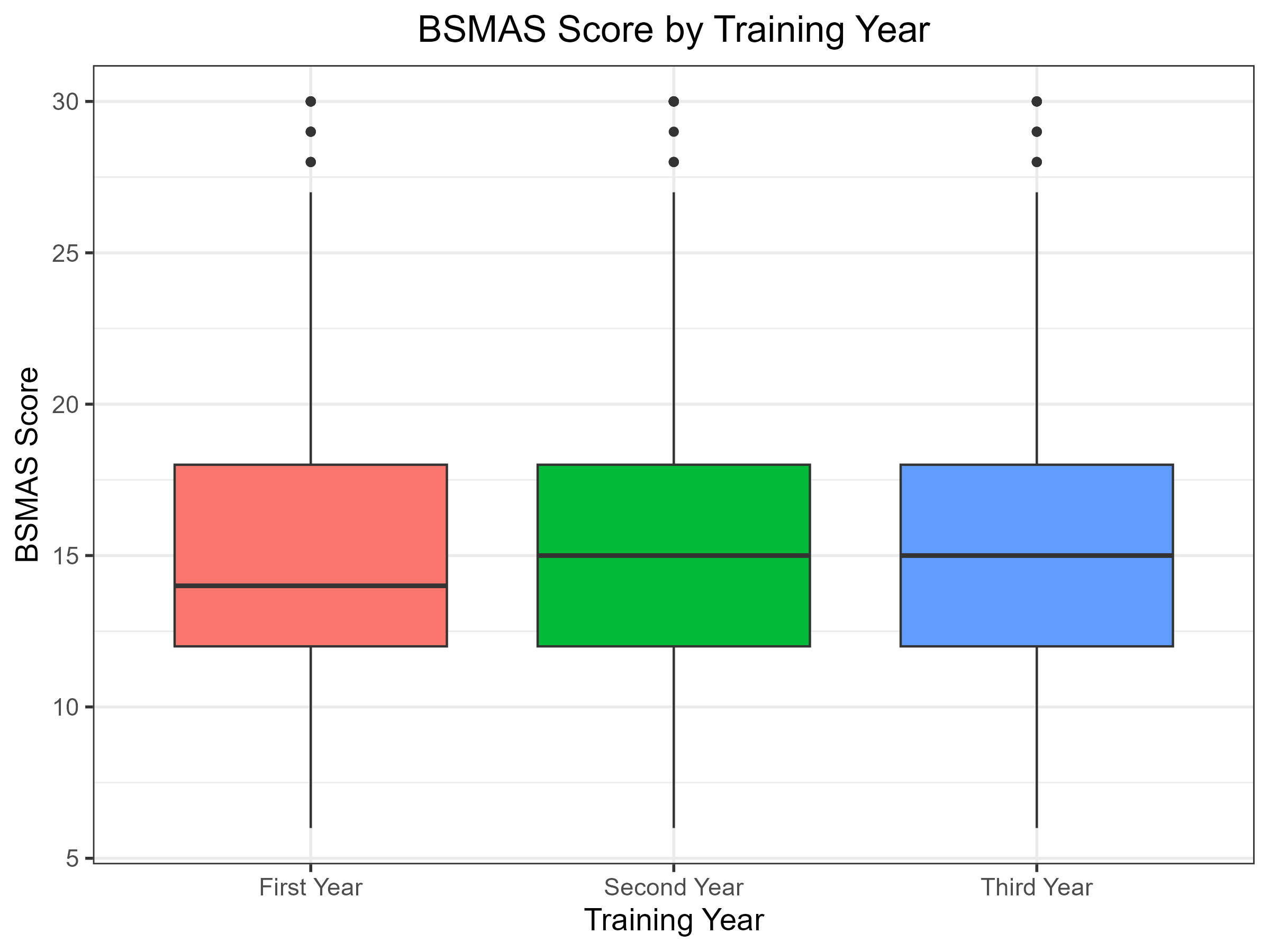


This boxplot shows the variation in BSMAS scores among first-, second-, and third-year resident physicians. The median, interquartile range, and outliers are displayed to highlight differences in social media addiction levels across training cohorts.

**Supplement Figure S4** **Forest Plot of Interaction Effects in Moderation Models**


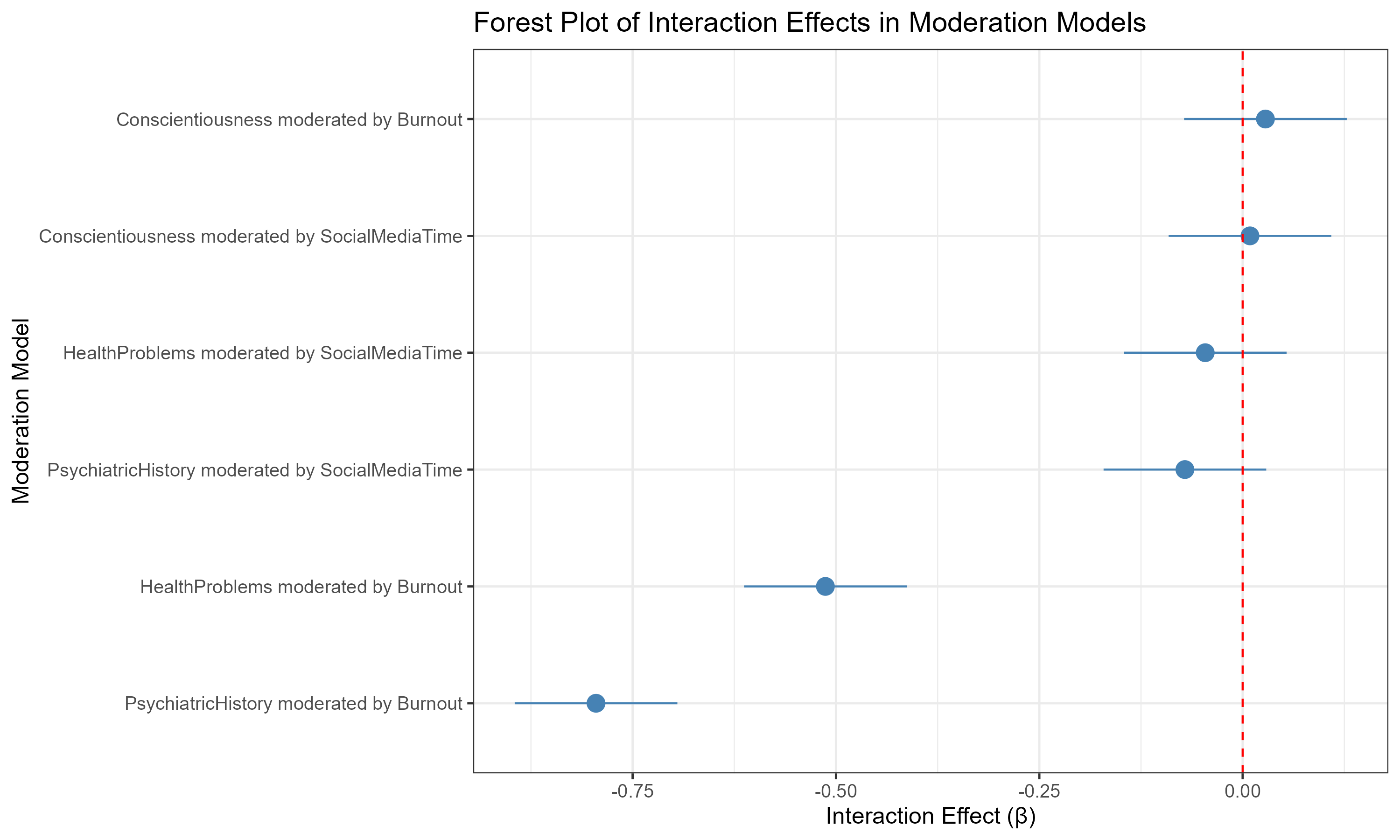


This figure presents a forest plot of interaction effects (β) in various moderation models. Each row represents a specific moderation model, with the interaction effect (β) and its confidence interval displayed. The red dashed line at β = 0 indicates the null effect. The results show that the interaction effects of "Psychiatric History moderated by Burnout" (β = -0.795, p < 0.001) and "Health Problems moderated by Burnout" (β = -0.513, p = 0.002) are statistically significant, while the other interaction effects are not significant.
