# Supplementary material for: Prevalence of Social Media Addiction and Associations With Usage Patterns, Burnout, and Health Conditions Among Medical Trainees in China: Cross-Sectional Study
Source: J Med Internet Res. 2026 May 4;28:e75675. doi: 10.2196/75675 (PMC13138709; doi:10.2196/75675)
Supplement: Checklist 1 [file jmir-v28-e75675-s002.docx]

# CHERRIES Checklist for Reporting Results of Internet E-Surveys

| **Item Category** | **Checklist Item** | **Explanation** | **Check** | **Notes for this project** |
| --- | --- | --- | --- | --- |
| Design | Describe survey design | Describe target population, sample frame. Convenience sample? | √ | Yes. the survey design has been clearly described and it was a convenience sample. |
| IRB approval | IRB approval | Mention whether the study has been approved by an IRB. | √ | Yes. IRB approval has been obtained and the approval number is provided. |
| Informed consent | Informed consent | Describe informed consent process (length, data storage, investigator, purpose). | √ | Yes. participants were fully informed about survey length, data storage, investigator, and study purpose before participation. |
| Data protection | Data protection | If personal information collected, describe protection mechanisms. | √ | No identifiable personal information was collected (contact details were optional). Data access was restricted to core research staff only. |
| Development and testing | Development and testing | How the survey was developed and tested for usability/technical functionality. | √ | The survey was developed by experienced psychologists using validated instruments. Pre-testing was conducted with 20 graduate students. |
| Recruitment | Open vs closed survey | Survey open to all visitors or restricted/password-protected sample. | √ | Open Survey |
| Recruitment | Contact mode | Was initial contact made online or via other methods? | √ | The survey was distributed online at the outset. |
| Recruitment | Advertising the survey | Where/how the survey was announced or advertised. | √ | Participants were recruited via WeChat, the most widely used social media platform in China. |
| **Item Category** | **Checklist Item** | **Explanation** | **Check** | **Notes for this project** |
| Survey administration | Web/E-mail | Type of survey (posted on website or sent via email). | √ | The survey was distributed via a WeChat link and hosted on a survey website; no email invitations were sent. |
| Survey administration | Context | Describe the website or environment where the survey was posted. | √ | The survey was hosted on Wenjuanxing (www.wjx.cn), the largest online survey platform in China. |
| Survey administration | Mandatory/voluntary | Was survey mandatory or voluntary? | √ | Voluntary |
| Survey administration | Incentives | Were incentives offered (monetary, prizes, feedback)? | √ | Yes. participants were offered a small incentive (5 RMB, <1 USD), which was unlikely to introduce response bias. |
| Survey administration | Time/Date | When was data collected? | √ | Data collection was conducted over approximately two weeks, from August 29 to September 10, 2024. |
| Survey administration | Randomization | Were items randomized or alternated to prevent bias? | × | The order of items was fixed; no randomization was applied. |
| Survey administration | Adaptive questioning | Use of adaptive questioning to reduce burden. | √ | Yes. adaptive questioning was implemented. |
| Survey administration | Number of items/screens | Number of items per page and number of pages. | √ | The questionnaire was structured into four sections: Section 1 (basic information: 15 categories), Section 2 (workload and lifestyle factors: 9 categories), Section 3 (mental health status: 3 standardized scales), and Section 4 (psychological characteristics: 9 categories). This comprised a total of 36 major question groups; including all matrix sub-items, the instrument contained 84 items in total. |
| Survey administration | Completeness check | Was consistency/completeness check implemented (e.g., JavaScript)? | √ | Key questions were mandatory, thereby ensuring completeness of data. |
| Survey administration | Review step | Could respondents review/change answers before submission? | √ | Yes. respondents could review or modify their responses their answers before submission. |
| Response rates | Unique site visitor | How unique visitors were defined (IP, cookies, etc.). | × | Not Applicable. |
| **Item Category** | **Checklist Item** | **Explanation** | **Check** | **Notes for this project** |
| Response rates | View rate | Ratio of unique survey visitors to unique site visitors. | × | Due to platform restrictions, data on the number of unique site visitors and survey visitors were not available; therefore, the view rate could not be calculated. |
| Response rates | Participation rate | Ratio of visitors agreeing to participate to those visiting survey page. | × | Due to platform restrictions, information on the number of visitors agreeing to participate was not available; therefore, the participation rate could not be reported. |
| Response rates | Completion rate | Ratio of participants finishing survey to those agreeing to participate. | × | Due to platform restrictions, the number of participants who agreed to participate versus those who completed the survey was not available; thus, the completion rate could not be determined. |
| Preventing duplicates | Cookies used | Were cookies used to prevent multiple entries? | √ | Yes. cookies were used. |
| Preventing duplicates | IP check | Were IP addresses used to detect duplicates? | √ | Yes. IP addresses were collected; however, since many participants responded under the same network (e.g., hospital public Wi-Fi), this item was not used to exclude cases. |
| Preventing duplicates | Log file analysis | Was log file analysis used to detect duplicates? | × | Due to platform restrictions, log file analysis could not be performed. |
| Preventing duplicates | Registration | In closed surveys, how duplicate entries were prevented. | × | Not Applicable. |
| Analysis | Handling incomplete questionnaires | Were incomplete questionnaires included in analysis? | √ | Only complete questionnaires (with all mandatory items completed) could be submitted. |
| Analysis | Atypical timestamps | Were questionnaires with atypical timestamps excluded? | √ | Yes. completion time was recorded, and questionnaires with implausibly short completion times were excluded. |
| Analysis | Statistical correction | Were weighting/propensity scores used to adjust sample bias? | × | The sample size was sufficiently large with a balanced gender distribution; therefore, no statistical correction was applied. |
